# A Multi-Model Assessment of the Air Quality and Health Impacts of U.S. Energy Decarbonization

**DOI:** 10.1021/acs.est.6c02914

**Published:** 2026-09-17

**Authors:** Jinyu Shiwang, Wei Peng, John Bistline, Jamil Farbes, Xinyuan Huang, Gokul Iyer, Jesse D. Jenkins, Eladio Knipping, Nicholas Mailloux, Erin Mayfield, Aranya Venkatesh, Alicia Zhao, Qianru Zhu

**Affiliations:** † School of Public and International Affairs, 6740 Princeton University, Princeton, New Jersey 08544, United States; ‡ Andlinger Center for Energy and the Environment, Princeton University, Princeton, New Jersey 08544, United States; § Department of Civil and Environmental Engineering, 6429 The Pennsylvania State University, University Park, State College, Pennsylvania 16802, United States; ∥ Watershed, San Francisco, California 94103, United States; ⊥ Massachusetts Institute of Technology, Cambridge, Massachusetts 02139, United States; # Evolved Energy Research, San Diego, California 92101, United States; ¶ Center for Global Sustainability, School of Public Policy, University of Maryland, College Park, Maryland 20742, United States; ∇ Department of Mechanical and Aerospace Engineering, Princeton University, Princeton, New Jersey 08544, United States; ○ 263283 Electric Power Research Institute (EPRI), Palo Alto, California 94304, United States; ⧫ Center for Sustainability and the Global Environment, Nelson Institute for Environmental Studies, 5228 University of Wisconsin−Madison, Madison, Wisconsin 53726, United States; †† Thayer School of Engineering, 3728 Dartmouth College, Hanover, New Hampshire 03755, United States

**Keywords:** net-zero transition, decarbonization pathways, sectoral mitigation allocation, energy system modeling, air pollution, health co-benefits

## Abstract

Net-zero transitions are expected to reduce ambient fine particulate matter (PM_2.5_) concentrations and the associated mortality burden. The magnitude and distribution of these health effects are substantially affected by energy technology choices and the resulting precursor emissions that contribute to ambient PM_2.5_. On the basis of a multi-model analysis using three leading energy system models for the United States (US-REGEN, GCAM-USA, and REPEAT), we demonstrate that a net-zero transition yields robust nationwide reductions in ambient PM_2.5_ concentrations and associated mortality. Under the net-zero scenarios, national PM_2.5_-attributable mortality rates in 2050 declined by 28–73% compared to 2017 across the three models. This result confirms substantial health benefits from reducing energy-sector pollution, despite concurrent population growth and aging trends that place upward pressure on mortality. Differences across the models result from how CO_2_ mitigation efforts are allocated across sectors and associated energy technology choices. For instance, the scale of carbon dioxide removal deployment in 2050 varies by a factor of three across the net-zero scenarios developed by the three models, influencing the amount of fossil fuel use that remains in a deeply decarbonized future and, consequently, the precursor emissions contributing to ambient PM_2.5_. By exploring sensitivity analyses that vary the projected emissions from non-energy activities such as wildfires, we also find that potential future increases in non-energy emissions can offset energy-sector health benefits.

## Graphical abstract

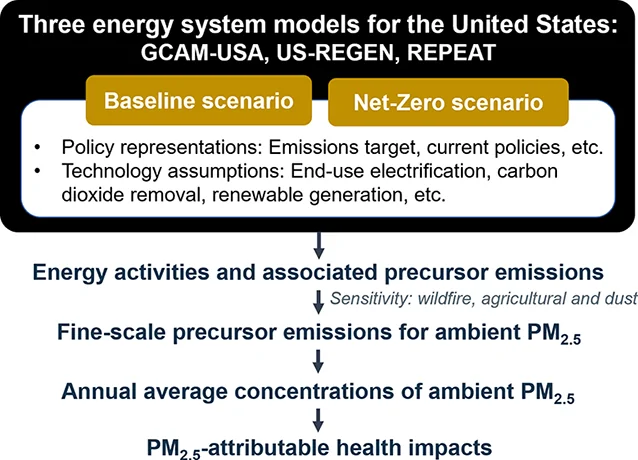

## 1 Introduction

A transition to a net-zero emissions economy (e.g., where remaining greenhouse gases (GHGs) are balanced by carbon dioxide (CO_2_) removals) is expected to reduce fossil fuel combustion and associated air-pollution health impacts. Net-zero transitions often rely on reducing CO_2_ and other GHGs from fossil fuel combustion, such as through coal plant retirement, scaling up renewable electricity, and end-use electrification. These changes can reduce co-emitted air pollutants, such as sulfur dioxide (SO_2_), nitrogen oxides (NOx), volatile organic compounds (VOCs), ammonia (NH_3_), and primary fine particulate matter (PM_2.5_). As these precursor emissions contribute to ambient PM_2.5_ concentrations through atmospheric transport and chemical transformation, net-zero transitions not only bring climate benefits but also improve air quality and reduce PM_2.5_-related health impacts.^1,2^ Globally, climate action consistent with a 2 °C target was estimated to avoid more than 13.5 million premature deaths from air pollution between 2020 and 2050.^3^ At the national or regional level, as countries transition toward net-zero emissions, improved air quality and reduced air-pollution-related health impacts are found for both developed economies and emerging markets, such as the United States,^4–6^ Europe,^7,8^ China,^9,10^ India,^11^ and other developing countries.^12,13^

Despite the same net-zero target, the health effects are not necessarily the same across all decarbonization pathways. The scale of air quality improvement depends on what and where fossil technologies are displaced and the resulting avoided emissions.^14,15^ For instance, net-zero emissions achieved with a heavy reliance on carbon dioxide removal (CDR) technologies allow for continued fossil fuel use even in a deeply decarbonized world.^16^ Some low-carbon strategies may also increase precursor emissions that contribute to ambient PM_2.5_. For instance, scaling up bioenergy use increases particulate emissions from direct combustion in the industrial and residential sectors,^17^ as well as NH_3_ emissions from the production of energy crops.^18^ In addition, net-zero pathways also differ in temporal patterns and cumulative impacts. Pathways with delayed mitigation or temporary overshoot may reach the same long-term climate target with substantially more cumulative GHG emissions as well as air pollution and health impacts.^19–21^

Given the complexity of the interconnected energy, pollution, and climate dynamics, the modeling community has developed an integrated modeling frameworks to couple energy system models with downstream impact assessment for air quality and health.^2,3,22,23^ Recent multi-model and scenario-ensemble studies have further examined air-quality and health outcomes across global decarbonization pathways and uncertainty drivers, including 1.5–2 °C mitigation pathways and future air-pollution damages.^24,25^ In the United States, a state-level assessment of net-zero pathways estimated $220–300 billion per year in avoided air-pollution damages from achieving the national net-zero goal,^4^ and an energy-transition health-impact analysis further estimated 67,000–81,000 avoided premature deaths.^6^

Yet, comparing existing modeling studies to distill robust and generalizable insights remains challenging. For the U.S. as an example, a suite of energy system models has been used to analyze the energy and emissions impacts of a net-zero transition. These models have fundamental differences in their core model structures (e.g., optimization models,^26–28^ market equilibrium models,^29,30^ and system dynamics models^31,32^ ), scope (e.g., global multi-sector models,^29,30^ subnational and sector-focused models^26–28,31,32^ ), and resolution (e.g., the number of technologies being represented and which technology choices are endogenously modeled). Therefore, these models can generate substantially different technology pathways to achieve the same CO_2_ or GHG target due to the differences in their representation of technology costs, infrastructure constraints, sectoral details, and policy implementations. These differences, in turn, lead to variations in the magnitude and sectoral distribution of precursor emissions, with important implications for air quality and health outcomes.

Recognizing the importance of these differences across energy system models, existing studies have evaluated the variations in energy and emission outcomes, but most multi-model studies do not expand the comparison to examine air quality and health outcomes^33–36^ or rely on a single energy model to estimate future health outcomes.^4–6^ One comparison study^23^ on pollution and health effects is limited to the national emission levels with a simplified source-receptor matrix^37^ to estimate the health effects. Such simplifications in impact assessment methods limit the ability to understand the subnational impacts, which are particularly important for air pollution and health outcomes given their inherently local nature. In addition, most energy modeling studies consider only emissions from the energy system, while non-energy emissions (e.g., NH_3_ from agriculture) interact with energy-sector emissions in ways that influence ambient PM_2.5_.^38,39^ Furthermore, non-energy emission sources such as wildfires are projected to play an increasingly prominent role in shaping future pollution levels.^40^ A comprehensive assessment of air quality and health impacts under net-zero pathways therefore requires an explicit consideration of how energy emissions interact with non-energy emissions.

*How do the net-zero pathways simulated by different energy system models influence the resulting air quality and health effects?* To address this question, we conduct a multi-model comparison study considering three energy system models with subnational detail for the U.S.: GCAM-USA,^30^ US-REGEN,^26,41^ and REPEAT^31,42^ (Figure 1). Using the technology- and region-specific emissions of CO_2_ and air pollutants simulated by each model, we then use the same impact assessment methods to evaluate ambient PM_2.5_-attributable health outcomes. This framework makes two key contributions: First, it helps identify robust insights across models and scenarios to deepen our understanding of the impact of the energy transition on human health. Second, this multi-model comparison approach helps identify key structural differences and scenario assumptions across energy system models that can shape downstream pollution and health effects.

**Figure 1:**
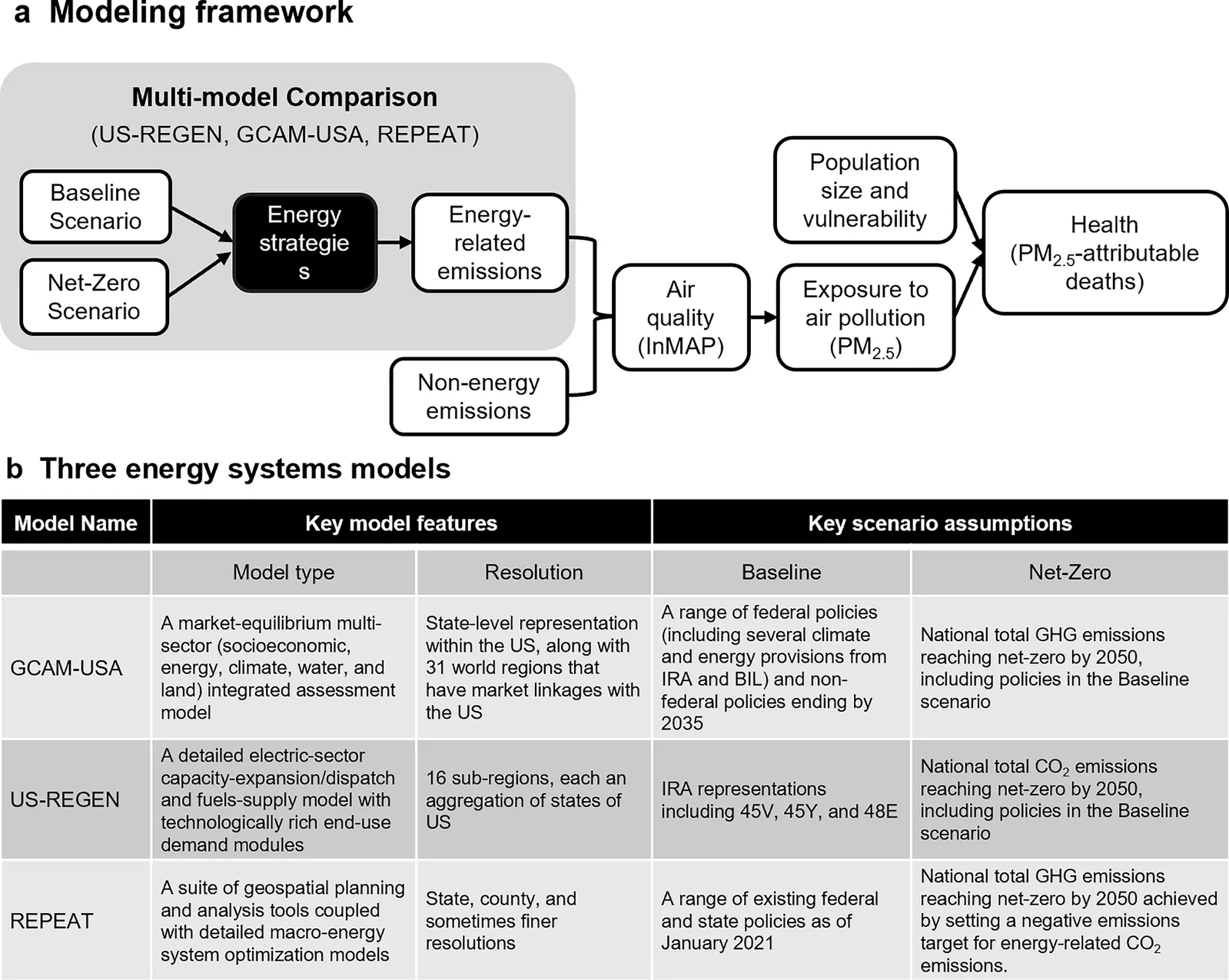
Modeling framework (a) and three energy systems models (b) used in this study. More details of our analytical methods and the three models are included in the Method section and Table S1. Here, policy abbreviations in panel b include the Inflation Reduction Act (IRA), the Bipartisan Infrastructure Law (BIL), the 45 V clean hydrogen production tax credit, and the 45Y and 48E clean electricity production and investment tax credits.

## 2 Materials and Methods

We develop an integrated modeling framework that links multiple energy system models with air quality and health impact assessments to evaluate the impacts of a Net-Zero transition across the U.S. (Figure 1a). We use three subnational energy system models, US-REGEN, GCAM-USA, and REPEAT, to generate mid-century scenarios, including a Baseline 2050 and a Net-Zero 2050 pathway (see key model differences summarized in Figure 1b). Emissions from the energy sectors are projected at the state or regional level by each model and then downscaled to 12 km × 12 km grids using sector-specific spatial patterns from the 2017 National Emissions Inventory (NEI2017). Exogenous non-energy emissions (e.g., agriculture, fugitive dust, and wildfire) are then combined with energy emissions for air quality modeling using the Intervention Model for Air Pollution (InMAP) to simulate annual average ambient PM_2.5_ concentrations at the county level. Finally, we combine the PM_2.5_ concentrations with SSP2 county-level population projections, baseline mortality rates, and national socio-demographic trends (GDP, population, age structure) to quantify the premature deaths attributable to ambient PM_2.5_ exposure. Below we describe each step in detail.

### 2.1 Three Energy System Models

We employ three widely used subnational energy system models: US-REGEN, GCAM-USA, and REPEAT. These models differ in main use case, temporal resolution and reporting years, model structure, treatment of existing assets, and technology representations, offering complementary perspectives on the dynamics governing human and energy systems. Below, we summarize key features of each model, with more information summarized in Figure 1b and Table S1.

#### 2.1.1 US-REGEN

U.S. Regional Economy, Greenhouse Gas, and Energy Model (US-REGEN) is a detailed energy system modeling framework developed by the Electric Power Research Institute (EPRI).^26^ It combines a detailed capacity-expansion and dispatch model of the U.S. electric power sector with a fuel supply model as well as a comprehensive end-use demand model for buildings, industry, and transportation. The power sector component co-optimizes generation, storage, and transmission investment with hourly dispatch decisions, providing high temporal and spatial resolution of technology competition and system operations. For instance, coal-plant retirement is determined endogenously within US-REGEN’s forward-looking, intertemporal optimization framework, which evaluates the long-term economics of continued operation of existing coal plants relative to alternative investments, including new natural-gas generation, renewables, and storage. The end-use model captures structural details across multiple sectors, including building stock, industrial process technologies, and vehicle fleets, allowing analysis of electrification, fuel switching, and efficiency. The fuels model characterizes a range of conventional and emerging low-carbon fuel supply pathways. The model is calibrated in 2015 and runs from 2015 to 2050 with five-year time steps. The model’s regional disaggregation (16 electricity market regions linked to load-serving areas) enables robust representation of renewable integration, storage, and transmission expansion. In US-REGEN, precursor emissions are estimated directly from detailed representations of generation technologies, fuel use, and end-use energy demand. The model reports sector-specific emissions at the regional level, with emission factors that reflect technology efficiency, pollution controls, and fuel switching under evolving policy and cost assumptions. These projections capture fine-scale changes in the power sector while maintaining consistency across other energy-related sectors.

#### 2.1.2 GCAM-USA

The Global Change Analysis Model (GCAM) is an integrated assessment model that links the energy, land, water, climate, and socioeconomic systems, developed by the Joint Global Change Research Institute (JGCRI).^43^ The global version of GCAM divides the world into 32 geopolitical regions, each with explicit representation of energy and socioeconomic systems, and includes 235 water basins and 384 land regions to capture water and land systems. GCAM tracks emissions and sinks of CO_2_, 16 other greenhouse gases, and multiple air pollutants. It is a market equilibrium model that solves for supply-demand balance every five years by adjusting prices, with technologies vintaged to allow existing stock to carry forward into future periods. The U.S. state-level version, GCAM-USA V8.2 (https://github.com/JGCRI/gcam-core/releases/tag/gcam-v8.2), disaggregates the energy and economy into all 50 states and the District of Columbia while retaining detailed representations of water and land sectors.^44,45^ GCAM-USA is calibrated in 2021 and runs from 2021 to 2050 in five-year time steps. The energy system includes both depletable resources (coal, oil, gas, uranium) and renewable resources (biomass, hydropower, solar, wind, and geothermal). State-level energy transformation processes, such as oil refining and power generation, are explicitly modeled. The electricity sector includes a full suite of technologies: fossil fuels with and without carbon capture and storage (CCS), bioenergy with CCS, renewables, and nuclear, used to supply state-level demands from buildings, transportation, and industry. GCAM-USA projects energy-related precursor emissions based on technology activity levels, fuel use, and emission coefficients that evolve with policy, efficiency, and fuel mix changes. These emissions are reported at the state level and vary with technology.

#### 2.1.3 REPEAT

The Rapid Energy Policy Evaluation and Analysis Toolkit (REPEAT) is a policy-focused, scenario-driven energy system modeling platform developed by Princeton University’s ZERO Lab in collaboration with Evolved Energy Research.^31,42^ It builds upon the Net-Zero America study framework and provides timely, independent analysis of U.S. decarbonization pathways under evolving federal and state policy landscapes.^31^ REPEAT runs from 2022 to 2050 with selected reporting years from 2022 through 2050 (e.g., 2022, 2024, 2026, 2028, 2030, 2032, 2035, 2040, 2050). REPEAT combines geospatial planning tools with an economy-wide energy systems intertemporal optimization framework with foresight to evaluate policy proposals and infrastructure investments. It can resolve siting and deployment of renewables, electrification of end uses, transmission expansion, and the role of CDR technologies (bioenergy with CCS, and direct air capture) under different policy scenarios. REPEAT generates air pollutant emissions by linking detailed sectoral energy demand with a supply-side macro-energy system model that tracks combustion, fuel switching, and technology deployment. Emissions are calculated using sector- and fuel-specific emissions coefficients and are reported at the state level for all energy and industrial sources. This structure allows REPEAT to capture the implications of rapid changes in renewable penetration, electrification, and fossil fuel displacement on future pollutant emissions.

### 2.2 Scenario Design

For each model, we construct two scenarios from now to 2050: Baseline scenario and Net-Zero scenario. The baseline considers existing trends and policies without a long-term decarbonization target, while the net-zero scenario imposes an ambitious decarbonization target to reach net-zero emissions by 2050. The three models are harmonized at the level of the broad scenario definitions, a current-policy baseline, and a 2050 Net-Zero pathway, but keep model-specific assumptions regarding technology and fuel costs, policy implementation, retirement rules, and investment decision frameworks. We therefore interpret the results as internally consistent model-specific pathways rather than fully technologically harmonized projections. The main results focus on 2050 because it is the target year when the net-zero constraints are designed to be fully binding, allowing us to compare long-term technology pathways across models at the end point of the transition. To address near-term pathway differences, we also conduct additional air-quality and health impact analyses for 2030 and report these results in the Supporting Information.

#### 2.2.1 US-REGEN

The baseline scenario explicitly represents recent federal policy commitments, including provisions from the Inflation Reduction Act (IRA) of 2022, including the 45 V hydrogen production tax credit, the 45Y and 48E credits expiring by 2035. Broadly, these measures serve to reduce fossil consumption in the medium term relative to a non-policy counterfactual case. The Net-Zero scenario adds a national target for economy-wide CO_2_ while also retaining the IRA provisions, again with 45Y and 48E phasing out by 2035. This design captures a transition pathway where the policy-driven deployment of low-carbon technologies is combined with economy-wide emissions caps to achieve net-zero CO_2_ by 2050. Also, the model tracks other non-CO_2_ GHG emissions in its baseline scenario, although it does not add a cap to these emissions in the net-zero scenario.

#### 2.2.2 GCAM-USA

The baseline scenario is derived from the current policies scenario from Zhao et al. 2024.^46^ This scenario includes existing federal policies, including several climate and energy provisions from the Inflation Reduction Act (IRA) and the Bipartisan Infrastructure Law (BIL), as well as nonfederal policies. In this study, we update this scenario under the GCAM-USA V8.2 model structure, including updating the calibration year from 2015 to 2021, and run the model from 2021 to 2050 with five-year time steps. Existing state-level renewable portfolio standards (RPS), corporate average fuel economy (CAFE) standards, and several IRA tax credits for renewable electricity generation, electric vehicles (EVs), and carbon capture and storage (CCS) are among the key policy drivers in this scenario. All of the policies extend until 2035, and we assume no further incentives beyond that. For the net-zero scenario, we impose a linear reduction trajectory for total GHG from 2025 to 2050 on top of those policy implications in the baseline scenario.

#### 2.2.3 REPEAT

The baseline scenario reflects a wide range of existing federal and state policies as of January 2021, including clean energy standards, efficiency measures, and emissions standards. It assumes gradual but incomplete decarbonization consistent with the currently legislated or announced actions. The Net-Zero scenario imposes a national cap on CO_2_ emissions from energy and industrial sectors, requiring reductions of 51% below 2005 levels by 2030 and net-zero total GHGs by 2050. While CO_2_ emissions are explicitly constrained, non-CO_2_ greenhouse gases follow exogenous trajectories consistent with deep decarbonization assumptions.

#### 2.2.4 Treatment of Base Year

Each model employs a different calibration year: 2015 for US-REGEN, 2021 for GCAM-USA, and 2022 for REPEAT. Although we compare the energy consumptions and conduct air quality and health impact assessments for each of these base-year outputs (Figures S1 and S2), we further consider 2017 as a common benchmark so that all future simulations are evaluated against a consistent historical baseline where the national primary energy and CO_2_ emissions are based on the U.S. Energy Information Administration (EIA),^47^ national final energy consumptions are based on the International Energy Agency (IEA),^48^ and fine-scale air pollutant emissions are based on the U.S. National Emissions Inventory (NEI 2017).^49^ Notably, while prior studies have found biases in U.S. National Emissions Inventories and identified potential missing or underestimated emissions,^50,51^ we choose NEI 2017 for base year air quality simulations, as it provides a complete, pre-COVID, spatially resolved, sector-specific emissions inventory for the major PM_2.5_ precursor emissions needed for this workflow.

#### 2.2.5 Treatment of Net-Zero Targets

The three models differ in how the net zero target is represented. In US-REGEN, the net-zero scenario constrains economy-wide CO_2_ emissions, while non-CO_2_ GHG emissions are tracked but not explicitly constrained. In REPEAT, the scenario is designed to be consistent with economy-wide net-zero GHG emissions by 2050: energy and industrial CO_2_ emissions are capped at a level consistent with overall net-zero GHGs after accounting for non-CO_2_ emissions from upstream energy, agriculture, and land use. In contrast, GCAM-USA has the capabilities to set a CO_2_-only target and a broader GHG constraint; therefore, for the main comparison with the other two models, we set a net-zero target for total GHG, and we then include a Net-Zero-CO_2_ sensitivity case to test whether the formulation of net-zero targets affects the PM_2.5_-attributable health conclusions. Analytically, we treat this as a parametric uncertainty related to the core scenario component, despite the real-world policy implications and debates around net-zero targets.^52^

### 2.3 Energy Sector Precursor Emissions

Each of the three models projected energy-related emissions under the baseline and net-zero scenarios but with different structures and sectoral coverage (Table S1). GCAM-USA reports state-level energy emissions by detailed sectors and technologies with additional national-level coverage of land use and agricultural emissions, but fugitive dust is not modeled. US-REGEN generates regional-level emissions covering energy, agriculture, and fugitive dust, with detailed sectoral resolution reflecting its capacity expansion and dispatch framework. REPEAT provides state-level energy-related emissions consistent with its scenario-driven optimization approach but does not explicitly represent agricultural, land-use, or fugitive dust emissions.

To enable a consistent comparison, we group energy-sector precursor emissions into four common categories: power, industry, residential commercial, and transportation (Table S2) for five major precursor emissions relevant for PM_2.5_ formation: SO_2_, NO_*x*_, NH_3_, VOCs, and primary PM_2.5_. We then apply a consistent static spatial downscaling method to downscale model-projected emissions to gridded emissions as input to air quality modeling. Specifically, we downscale state- or regional-level, annual energy-related precursor emissions from US-REGEN, GCAM-USA, and REPEAT to a 12 km × 12 km grid based on the spatial patterns in NEI 2017.^49^ The downscaling is performed for four aggregate energy-relevant categories (see Table S3 for mapping NEI sectoral emissions to these four categories) and five major precursor emissions relevant for PM_2.5_ formation. For each pollutant and category, we first aggregate gridded NEI emissions to the state level for GCAM-USA and REPEAT and to the regional level for US-REGEN. We then compute the share of each grid cell in the state or region’s total emissions to derive a downscaling factor. These factors are assumed to remain constant across all years, thereby preserving within-state spatial patterns while scaling emissions in proportion to each model’s projections. This procedure ensures comparability across models despite differences in the original spatial resolutions.

### 2.4 Non-energy Emissions

In addition to energy-related emissions, we account for three major non-energy sources: wildfire, agricultural, and fugitive dust emissions. We hold non-energy emissions at the 2017 level in the main baseline and Net-Zero 2050 scenarios. This approach helps to tease out the effects of energy system transformations on pollution and health outcomes.

To consider the potential effects of rising non-energy emissions, we perform sensitivity analyses where we use high projections of wildfire, agriculture, and fugitive dust emissions. For wildfire emissions, we use projections of primary PM_2.5_, the major pollutant from wildfires, from the U.S. Forest Service dataset,^53^ which has been applied in prior studies of global health and air quality impacts.^54^ This dataset provides CESM-based projections of climate- and human-driven fire emissions for multiple species at a global 0.9 ° × 1.25 ° resolution, including both historical years (2000–2010) and future periods (2050, 2100) under various SSP-RCP scenarios. For agriculture emissions, we use projections of NH_3_, the major pollutant from agricultural activities, from a previous study.^55,56^ This dataset provides global NH_3_ emissions from the CAMEO process-based model at a global 2.5° × 1.27° resolution, available for historical (2000–2014) and future periods (2015–2100) under various SSP-RCP scenarios. For both global datasets, we extract emissions over the continental United States, and re-grid them to match the resolution of NEI 2017. We then re-grid the wildfire and agricultural emissions to the same 12 km × 12 km grid, thereby harmonizing spatial representation across all sources. For fugitive dust emissions, we use the increasing projected regional emissions from the US-REGEN team and apply the same static downscaling method to downscale them to the gridded level as we did for energy sectors. Considering the different assumptions used in the datasets, we choose the higher projections of three non-energy emission sources (SSP1-RCP4.5 for fire, SSP5-RCP8.5 for agriculture, and US-REGEN projections for dust) to build our “High non-energy emissions” case. The summary of national total non-energy emissions is shown in Table S4.

### 2.5 Air Quality Modeling

For each scenario, we use the downscaled emissions to simulate the annual average ambient concentrations of PM_2.5_ using the Intervention Model for Air Pollution (InMAP), a reduced-complexity, national-scale air quality model.^57^ InMAP estimates ambient PM_2.5_ from precursor emissions of SO_2_, NO_X_, NH_3_, VOCs, and primary PM_2.5_. For energy-related emissions, we use the projected and downscaled emissions from US-REGEN, GCAM-USA, and REPEAT at a 12 km × 12 km resolution. For non-energy emissions, we directly used the gridded NEI 2017 emissions in the main analysis and the projected and re-gridded emissions as described above.

Methodologically, InMAP is built upon the general reaction–advection–diffusion equation:

1 $$\frac{\partial C_{i}}{\partial t}=-\nabla \cdot \left(\mathrm{u⃗}C_{i}\right)+\nabla \cdot \left(K\nabla C_{i}\right)+R\left(C_{i}\right)+E_{i}-D_{i}$$

where C_i_ is the concentration of modeled species (i), u⃗ is the wind vector driving advection, K is the turbulent diffusion tensor, R­(C_i_) represents the net chemical production and loss of species (i) as a function of the vector of modeled species concentrations (C_i_), E_i_ is the emission flux, and D_i_ is the removal flux from wet and dry deposition. The chemical transformation term represents the conversion of precursor emissions, including SO_2_, NOx, NH_3_, and VOCs, into PM_2.5_ components such as sulfate, nitrate, ammonium, and secondary organic aerosol while also accounting for chemical loss processes in the reduced-form representation.

InMAP assumes a steady-state solution (∂C_i_/∂t = 0), so modeled concentrations represent annual-average equilibrium responses to emissions, transport, chemistry, and removal. Rather than dynamically simulating meteorology and atmospheric chemistry for each scenario, InMAP uses preprocessed annual-average parameter fields derived from WRF-Chem, including annual-average wind speed and direction, vertical mixing coefficients, temperature, wet and dry deposition rates, and chemical reaction rate constants. These fixed parameter fields allow InMAP to efficiently estimate annual-average PM_2.5_ responses while retaining simplified representations of the dominant transport, transformation, and removal processes. A distinctive feature of InMAP is its variable-resolution computational grid, which dynamically allocates finer resolution in densely populated regions (down to 1 km × 1 km in urban areas) and coarser resolution in sparsely populated regions (up to 48 km × 48 km), thereby efficiently capturing spatial gradients in exposure while minimizing computational burden.

Model evaluation against full chemical transport models (CTMs) has shown that InMAP reproduces annual-average, population-weighted changes in PM_2.5_ with good accuracy, achieving an *R* ^2^ of ∼0.9 and a mean fractional bias of ∼−17%.^58^ These metrics fall within accepted bounds for policy-relevant air quality modeling.^57^ Although InMAP does not resolve hourly meteorological variability or fully detailed atmospheric chemistry, it has been widely used to couple with energy modeling to provide a rapid assessment of the long-term PM_2.5_-attributable health impacts of energy transition.^22,58,59^

### 2.6 Health Impact Assessment

Here, we conduct health impact assessments in each of 3108 counties across the continental U.S. To quantify the county-level exposure to ambient PM_2.5_, we first estimate the county-average exposure level based on the InMAP-simulated PM_2.5_ concentrations that are at varying resolutions from 1 km × 1 km to 48 km × 48 km. For large counties, we take the average of the PM_2.5_ concentrations from multiple InMAP gridded data points. For small counties, we use the same PM_2.5_ concentrations for all the counties located within one InMAP grid.

We then quantify all-cause premature deaths attributable to long-term exposure to ambient PM_2.5_ in each county using Pope et al.’s log–linear health-impact function^60^ from BenMAP:

2 $$\Delta Y=AF\cdot Y_{0}\cdot Pop=\left(1-e^{-\beta \cdot \Delta PM}\right)\cdot Y_{0}\cdot Pop$$

The detailed information for each component is presented in Table 1.

**Table 1:** Summary of Input Assumptions and Data for the Health Impact Assessment

variable	definition	data source
Y_0_	All-cause baseline mortality rate	Projections from the Environmental Benefits Mapping and Analysis Program (BenMAP)^61^ are reported in five-year increments from 2015. Since our base year for emissions and exposure is 2017, but mortality data are only available in 2015 bins, here, we use 2015 mortality rates as a proxy for 2017.
pop	County-level population	For 2017, we use projections from the US Census Bureau census block data.^62^
		For 2030 and 2050, we get county-level SSP2 projections from the Socioeconomic Data and Application Center (SEDAC).^63^ We then multiply a scaling factor for each model to make the national total population consistent with each model’s population scale.
β	Changes in mortality risk resulting from changes in the PM_2.5_ exposure level	The central value and 95% CIs are derived from Pope et al. 2019.^60^ Here, we use the same estimate for 2017 and 2050, where RR = 1.12 per 10 μg/m^3^ PM_2.5_ (95% CI: 1.08–1.15). We also consider the Global Exposure Mortality Model (GEMM)^64^ as a sensitivity analysis.
ΔPM	County-level PM_2.5_ concentration	County-level PM_2.5_ concentrations are averaged from gridded concentrations simulated by InMAP.

We report both the total PM_2.5_-attributable deaths and population-normalized PM_2.5_-attributable death rates. Death rates are calculated as attributable deaths divided by the exposed population and are reported as the number of deaths per million people. We use death rates when comparing changes across time and across models because the population size differs between 2017 and 2050 and varies slightly across model-specific socioeconomic assumptions.

### 2.7 Decomposition Analysis

To decompose the impacts on CO_2_ emissions, we consider three factors: (1) effects of socio-economic changes, (2) effects of energy system changes in the Baseline scenario, and (3) effects of the energy system changes to reach Net-Zero emissions. We begin with national CO_2_ emissions in 2017 as the reference level. We first apply the Kaya identity^65^ (eq 3) to quantify the effects of socio-economic changes in 2050 compared to 2017 across three models:

3 $${CO}_{2}\;emissions=P\times \frac{G}{P}\times \frac{E}{G}\times \frac{F}{E}$$

Specifically, we hold the 2017 energy intensity (E/G) and carbon intensity (F/E) consistent in 2050, so the emission increase is only from the population changes (P) and GDP per capita (G/P). Next, we show the reductions in CO_2_ emissions in each model’s Baseline scenario, which captures the baseline energy transitions and technological improvements. Finally, we show the additional CO_2_ reductions to reach the Net-Zero target compared to the Baseline scenario, which captures the effects of energy system change due to the Net-Zero transition.

To decompose the effects of different drivers on the PM_2.5_-attributable death rates, we consider four factors: (1) effects of socio-demographic changes, (2) effects of energy system changes in the Baseline scenario, (3) effects of the energy system changes to reach Net-Zero emissions, and (4) effects of the non-energy emission projections in the Net-Zero scenario. We start with emissions from the 2017 NEI inventory, combined with 2017 population and baseline mortality data, to establish the reference estimate of PM_2.5_-attributable death rates. In the next step, we hold emissions fixed at 2017 levels but update the socio-demographic inputs (population size, age structure, baseline mortality rates) to their 2050 projections, thereby quantifying the effect of socio-demographic changes. We then introduce the 2050 baseline energy system emissions (while keeping socio-demographics at 2050 levels), which captures the additional effect of energy and technology transformations expected under each model’s Baseline scenario. In the following step, we replace the 2050 Baseline emissions with those from the 2050 Net-Zero scenarios, yielding the additional effects of the energy system changes to reach Net-Zero emissions. Finally, we conduct a sensitivity analysis to compare the Net-Zero outcomes under constant 2017 non-energy emissions to those under projected increases in wildfire, fugitive dust, and agricultural sources, thereby highlighting the sensitivity of health benefits to assumptions about non-energy emissions. This framework ensures that the total changes in health outcomes between 2017 and 2050 can be attributed to specific components: socio-demographic inputs, baseline energy system dynamics, additional energy system changes required for reaching net-zero targets, and the role of non-energy emissions.

## 3 Results and Discussion

### 3.1 Impacts of Energy Transition on National CO_2_ and Health

In the baseline scenarios, we find consistent macro-scale trends across the three models where the national total GHG emissions and PM_2.5_-attributable death rates decline from 2017 to 2050 (Figures 2 and 3). The decrease in total GHG emissions is large (by 38–69% across models), whereas the reduction in national PM_2.5_-attributable death rates is modest (by 17–36%). Such patterns are driven by two competing forces: socioeconomic changes, especially population and GDP growth (Tables S5 and S6), drive up energy demand and associated emissions of both GHG and air pollutants, whereas the increasing share of cleaner sources in the energy mix drives down the emissions. The net effect of these two forces is a reduction in GHG over time, as the changes in the fuel mix and energy intensity more than offset the effects of demand growth. However, these substantial reductions in GHG emissions do not translate proportionally to improvements in air quality and health impacts. The reduction in PM_2.5_-attributable deaths is smaller since the demographic changes directly affect the size of the exposed population (e.g., population growth, see more in Table S5) and the nonlinear atmospheric processes and exposure–response relationships further dampen the effects.

**Figure 2:**
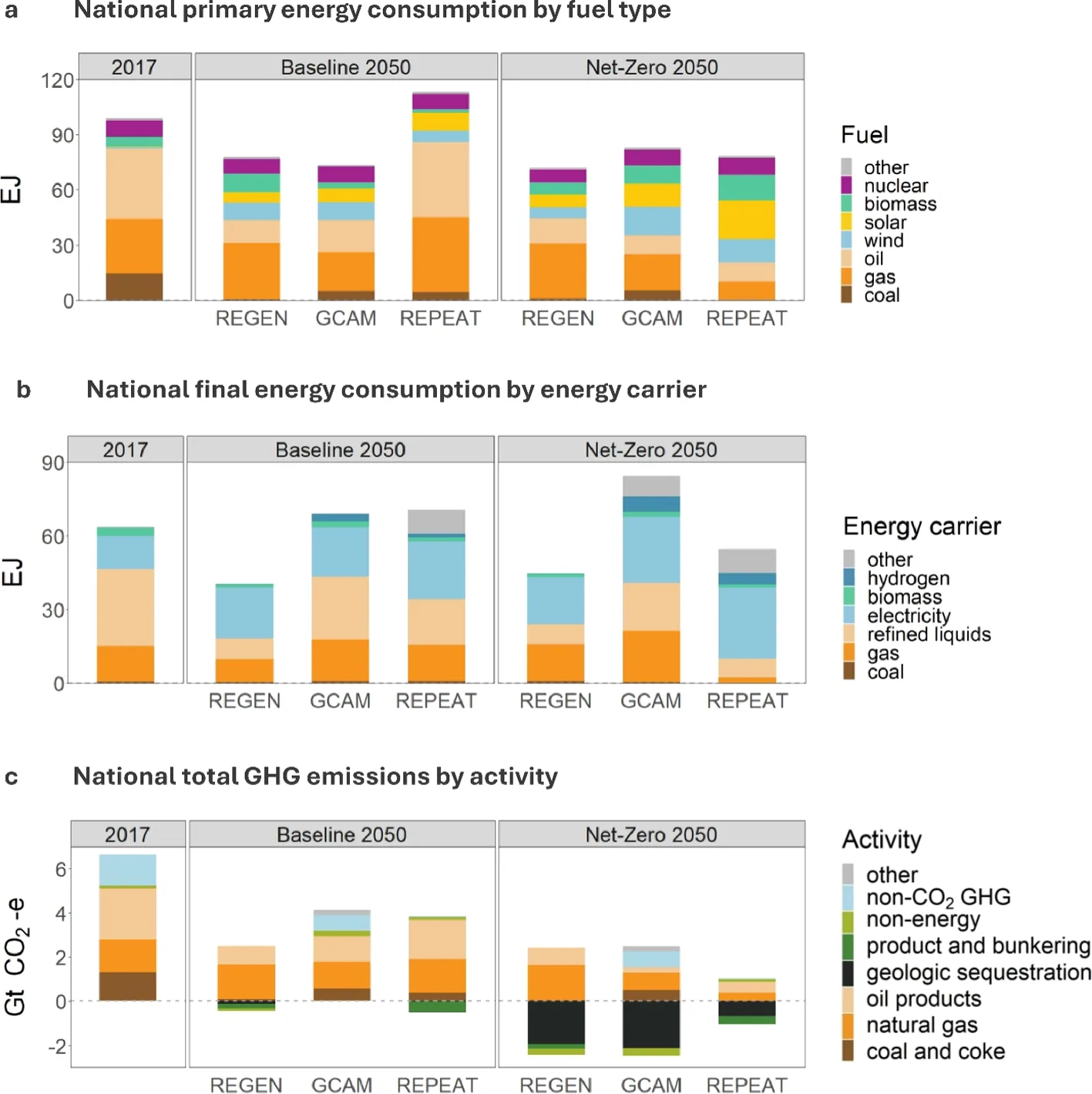
National primary energy use, final energy use, and GHG emissions. Panels show results for historical data (2017) and model projections for Baseline 2050 and Net-Zero 2050 scenarios using the US-REGEN, GCAM-USA, and REPEAT models, respectively. 2017 data for primary energy consumption and CO_2_ emissions are from the U.S. Energy Information Administration (EIA),^47^ non-CO_2_ GHG emissions from the U.S. Environmental Protection Agency (EPA),^67^ and that for final energy consumption from the International Energy Agency (IEA).^48^ Here GHG emissions for GCAM-USA and REPEAT include both CO_2_ and non-CO_2_ emissions, while US-REGEN only include CO_2_ emissions. All three models cover emissions from energy sectors and non-energy sectors.

**Figure 3:**
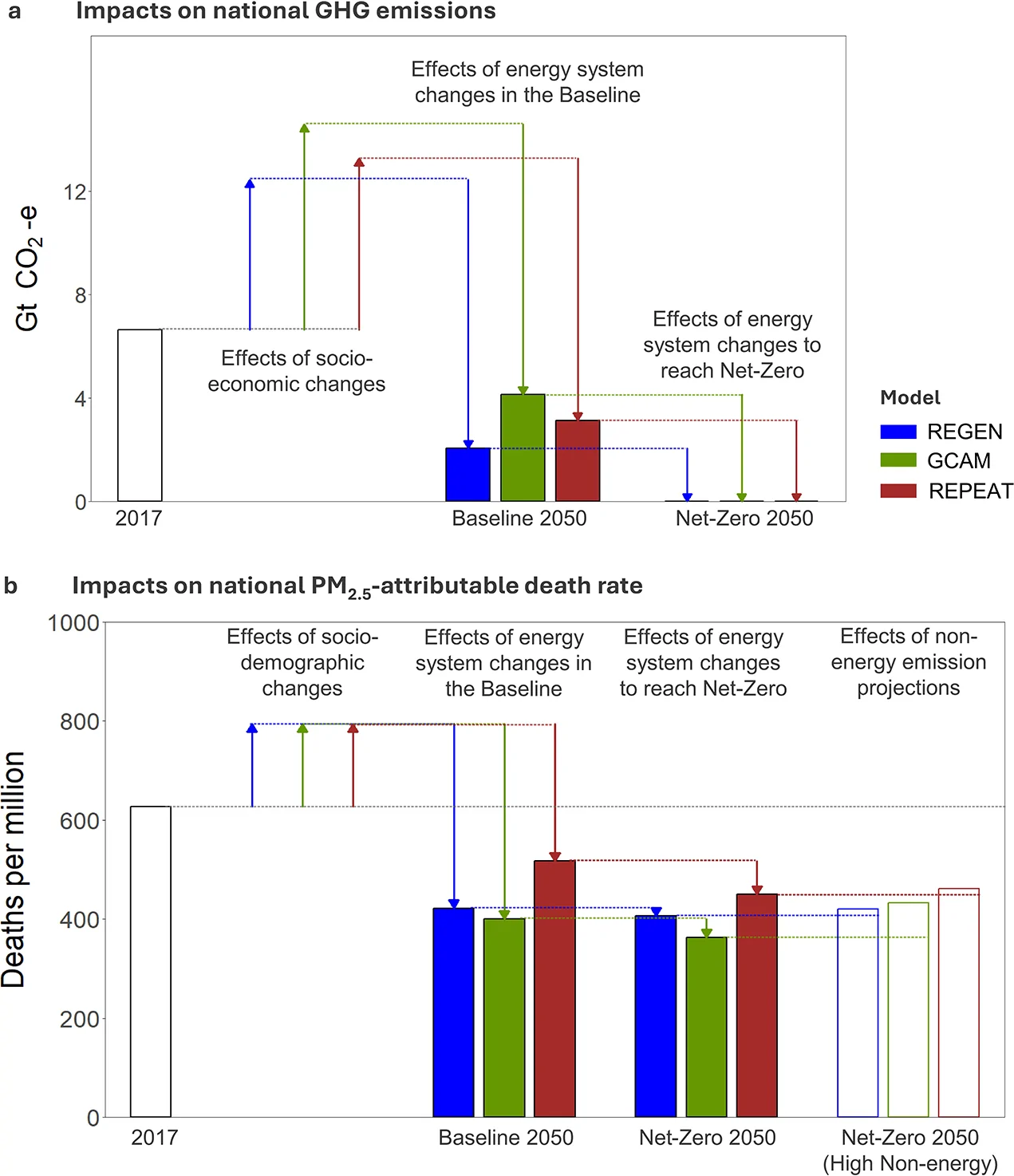
Effects of socioeconomic and technological factors on national GHG emissions and health effects. Panels show the relative contributions of socio-demographic, energy system changes, and non-energy factors to the differences in national GHG emissions and PM_2.5_-attributable death rates (per million deaths) between 2017, Baseline 2050, and Net-Zero 2050 scenarios. Bars represent model-specific estimates for 2017 and the two 2050 scenarios, and arrows illustrate the effects of each factor, including the direction and the magnitude. The effects of non-energy emissions are quantified by applying high projected emissions from wildfire and agricultural activities from other datasets^53,56^ and fugitive dust emissions from US-REGEN. The three colors represent the three models: US-REGEN (blue), GCAM-USA (green), and REPEAT (brown).

Despite a consistent declining trend over time, noticeable differences still exist across models for their Baseline scenario projections, particularly in the composition of primary and final energy use (Figure 2a,b). In the Baseline 2050 projections, all three models show a substantial shift away from coal and toward cleaner energy sources; however, the pace and structure of this transition vary. US-REGEN’s comparatively rapid decline in coal use is primarily driven by endogenous retirement decisions in its perfect-foresight, intertemporal power-sector optimization, which evaluates the long-term economics of existing coal generation relative to new natural-gas generation, renewables, and storage under assumptions about fuel prices and current policies, including IRA incentives. US-REGEN also projects relatively lower final energy demand (36% decrease from 2017 to 2050) compared to GCAM-USA and REPEAT, reflecting a combination of slower GDP growth projections (Table S6) and greater clean energy transition drivers in its Baseline scenario, including extensive transportation electrification.^66^ In contrast, GCAM-USA and REPEAT maintain relatively high levels of final energy consumption in 2050, but differ markedly in their primary energy consumption. GCAM-USA projects a substantial reduction in primary energy (26% decrease from 2017 to 2050), reflecting efficiency gains and electrification that reduce conversion losses. However, REPEAT maintains higher primary energy use, due to an increased deployment of renewable electricity, while fossil fuel use is not phased out, particularly natural gas (76% increase from 2017 to 2050).

Under the Net-Zero scenarios, the net GHG emissions of GCAM-US and REPEAT and the net CO_2_ emissions of US-REGENare expected to reach zero in all models in 2050. However, different models offer different least-cost technology pathways to achieve net zero emissions: the REPEAT model emphasizes phasing out unabated fossil fuels and accelerating end-use electrification, while GCAM-USA and US-REGEN lean more heavily on CDR technologies that involve geological CO_2_ sequestration (Figure 2c). This also explains the divergent patterns in energy consumption (Figure 2a,b): the increase in energy consumption under Net-Zero relative to Baseline scenario in US-REGEN and GCAM-USA is largely due to the additional energy needed from CDR technologies (in particular, the increasing gas use and electricity expansion in GCAM-USA), whereas the substantial reduction in REPEAT shows its strong reliance in fossil phase-out to reach net-zero. These differences arise from a range of factors, including model-specific socioeconomic pathways, policy representations, and technology and fuel costs, as well as how technology choice is modeled in the model’s decision-making frameworks. For instance, the divergent coal outcomes reflect three key structural differences across the models. First, GCAM-USA represents coal retirement primarily through state-specific, vintage-based exogenous trajectories, whereas US-REGEN and REPEAT allow additional retirements to respond endogenously to economic and policy conditions. Second, US-REGEN and REPEAT use perfect-foresight intertemporal optimization, but its implications differ: foresight accelerates coal retirement in both US-REGEN scenarios under current-policy incentives, while in REPEAT, it is most consequential when the model anticipates the binding net-zero emissions constraint; GCAM-USA instead uses a recursive-dynamic framework without such foresight. As a result, GCAM-USA’s economy-wide net-GHG constraint does not require a technology-specific coal phase-out. Existing coal vintages can remain according to their retirement trajectories, while coal technologies with CCS become available as a mitigation option, and residual emissions from the remaining coal fleet can be balanced by CDR elsewhere in the energy system.

The number of PM_2.5_-attributable death rates in the Net-Zero scenarios also differs across models (Figure 3b, with absolute deaths in Figure S3). In the Net-Zero scenario, GCAM-USA projects the lowest PM_2.5_-attributable death rate (∼362 per million (95% CI: ∼248–∼445)), whereas REPEAT projects the highest (∼451 per million (95% CI: ∼309–∼553)), with US-REGEN falling in between (∼407 per million (95% CI: ∼279–∼499)). These differences cannot be simply explained by aggregate fossil fuel use alone. Instead, they reflect differences in the composition and sectoral distribution of residual air pollutant emissions across models, which play a leading role in shaping population exposure and health outcomes. For example, GCAM-USA retains more residual fossil fuel use to support CDR, but it projects larger reductions in some industrial precursor emissions, whereas REPEAT achieves large power-sector emission reductions but leaves higher residual emissions in some transportation and industrial sources.

Notably, the pollution and health results are also affected by the emissions from non-energy sectors. When applying high projected emissions from wildfire, fugitive dust, and agricultural activities, we find that PM_2.5_-attributable death rates under the net-zero scenarios rise by 3–16% across all three models (Figure 3b). These increases do not offset the overall reduction in health benefits relative to those of 2017, but they narrow the health benefits from energy-sector decarbonization. This result indicates that future non-energy emissions are important uncertainties when interpreting the long-term health co-benefits of net-zero pathways.

### 3.2 Impacts of Sectoral Strategies on Precursor Emissions

To further understand the impacts on air quality and health, here, we unpack the impacts of underlying sectoral and technology choices on precursor emissions (Figure 4). We highlight two key findings. First, the cross-model differences within the energy sector are large. In the Net-Zero scenarios, REPEAT achieves substantial reductions in fossil fuel use, as shown in Figure 2; however, these reductions are concentrated primarily in the power sector. This leads to large declines in power-sector SO_2_ emissions (Figure 4b) but more limited reductions in transportation- and industrial-sector precursor emissions, especially for NO_*x*_ and VOC (Figure 4a,c). In contrast, US-REGEN projects pronounced reductions in transportation-related emissions, reflecting extensive end-use electrification and reduced reliance on oil-based fuels. Finally, GCAM-USA exhibits larger reductions in industrial-sector emissions compared to the other two models, which increasingly dominate residual air pollution under Net-Zero pathways.^68–70^ These cross-model differences in precursor emissions contribute to the national differences (Figure 3b) and subnational variations in the pollution and health effects (Figure 5).

**Figure 4:**
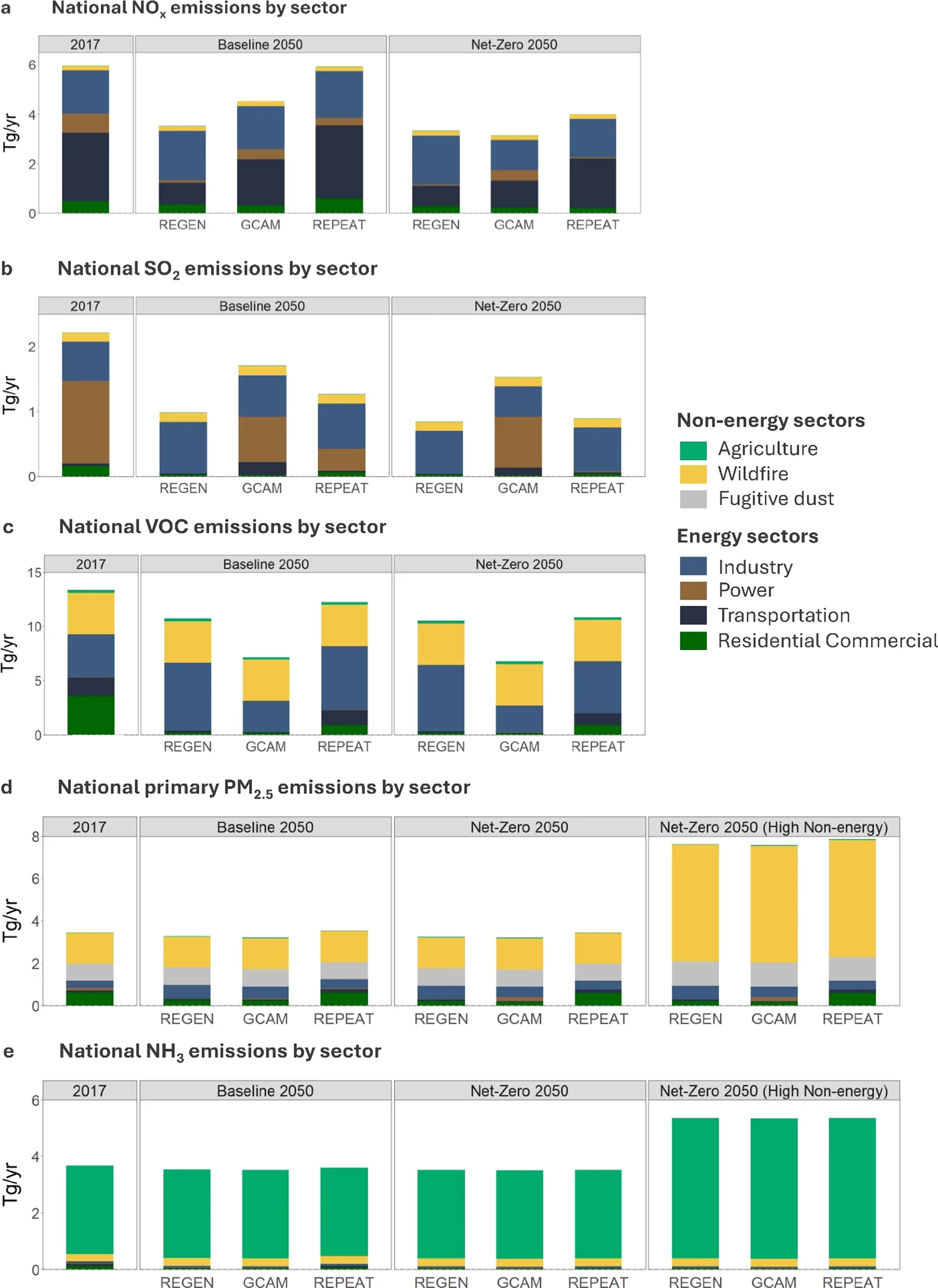
National precursor emissions by sector. Panels show the national precursor emissions in 2017 and projections for Baseline 2050 and Net-Zero 2050 scenarios using the US-REGEN, GCAM-USA, and REPEAT models, respectively. Panels a–e show the emissions of nitrogen oxides (NO_*x*_), sulfur dioxide (SO_2_), volatile organic compounds (VOC), primary fine particulate matter (PM_2.5_), and ammonia (NH_3_), respectively. Historical data for 2017 are from the U.S. National Emissions Inventory (NEI2017).^49^ In the main “Baseline 2050” and “Net-Zero 2050” scenarios, the non-energy emissions are kept at the 2017 levels, while “Net-Zero 2050 (High Non-energy)” includes a high projection of non-energy emissions from other datasets.

**Figure 5:**
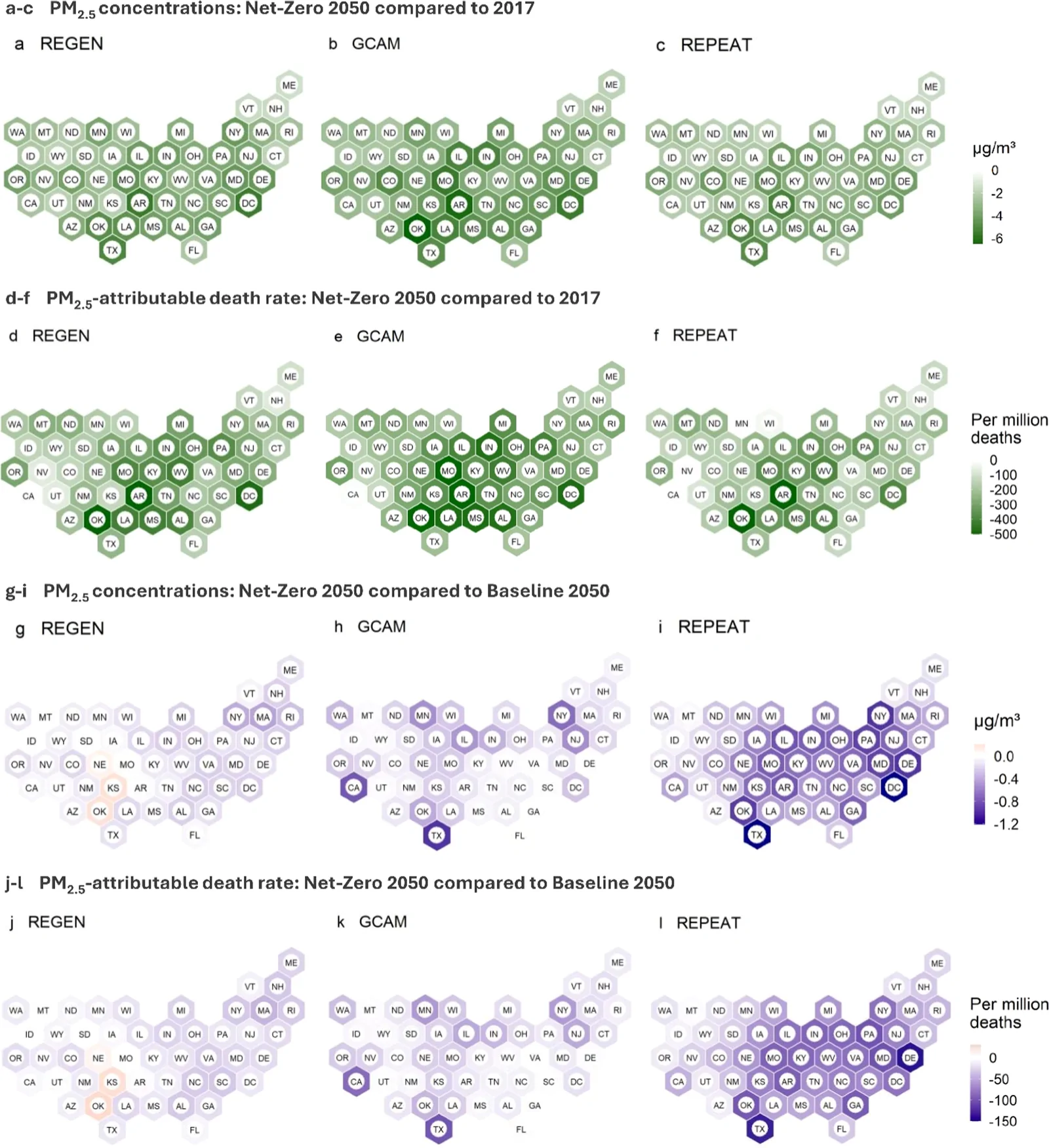
State-level changes in PM_2.5_ concentrations and PM_2.5_-attributable death rates. Panels show changes in annual average PM_2.5_ concentrations (panels a–c, g–i) and PM_2.5_-attributable death rates (panels d–f, j–l) across U.S. states under Net-Zero 2050 scenarios relative to 2017 and Baseline 2050 for the US-REGEN, GCAM-USA, and REPEAT models, respectively. Panels a–f show reductions in PM_2.5_ concentrations and associated mortality between Net-Zero 2050 and 2017, while panels g–l show the additional improvements achieved when transitioning from Baseline 2050 to Net-Zero 2050. Deeper colors indicate larger benefits, with green representing benefits relative to 2017 and purple representing additional benefits relative to Baseline 2050.

Second, the relative contribution of energy versus non-energy emissions varies substantially across emission types (Figure 4d–e). For instance, NO_*x*_ and SO_2_, as precursor emissions to secondary PM_2.5_, are almost exclusively emitted from energy sources, whereas NH_3_, another precursor emission to secondary PM_2.5_, is primarily emitted from agricultural sources. In contrast, for primary PM_2.5_ emissions, the contribution from the energy sectors is only 34% in 2017 and 29–34% under the Net-Zero 2050 scenarios across three models. Wildfire and fugitive dust are also major sources of primary PM_2.5_ emissions, and their relative importance could increase substantially in the future. This is particularly true under Net-Zero 2050 scenarios with high wildfire projections, where only 13–15% of primary PM_2.5_ emissions are attributable to energy sectors. Our findings thus underscore the need to combine both energy and land-use and management strategies to effectively manage and reduce future air pollution.

### 3.3 Subnational Variations in Pollution and Health Impacts

With a net-zero transition, subnational changes over time exhibit similar spatial patterns across the models. Because energy-sector emissions are downscaled using static 2017 spatial patterns, we focus the main interpretation on state-level patterns (Figure 5) and retain county-level maps in the Supporting Information as illustrative spatial results (Figure S4). When comparing Net-Zero 2050 with 2017, all three models project broadly similar spatial distributions of improvements, with the largest reductions in PM_2.5_ concentrations and PM_2.5_-attributable death rates occurring in the central and southern U.S., including Texas, Oklahoma, and Arkansas. Although the spatial patterns are broadly consistent, the magnitude of improvements varies across models, which aligns with the scale of the national-level changes. For both PM_2.5_ concentrations and PM_2.5_-attributable death rates, GCAM-USA consistently projects the largest reductions from 2017 to 2050 in most states, whereas US-REGEN and REPEAT exhibit more modest improvements.

However, when comparing the Net-Zero scenario to the Baseline scenario, which is often used to quantify the co-benefits of decarbonization, larger cross-model differences are observed (Figure 5g–l). On the basis of this comparison, only REPEAT shows consistent decreases in PM_2.5_ concentrations and PM_2.5_-attributable death rates across all states, since REPEAT relies heavily on phasing out fossil fuels to reach net-zero emissions. In contrast, GCAM-USA and US-REGEN project only modest reductions in the net-zero scenario compared to the baseline, since their baseline scenario already assumes large declines in pollution, leaving limited potential for further reductions through deep decarbonization. In fact, slightly higher pollution and health impacts are observed in some states under the net-zero scenario than under the baseline. For instance, for US-REGEN, some southwest states (e.g., Kansas, Nebraska, Oklahoma, and South Dakota) show an increase in pollution (Figure 6d) due to increasing industrial emissions when supporting CDR technologies such as direct air capture. For GCAM-USA, some southeast states (e.g., Florida) increase their emissions slightly under Net-Zero due to increased power-sector emissions (especially SO_2_ and primary PM_2.5_, see Figure S5) associated with large-scale deployment of bioenergy with CCS.

**Figure 6:**
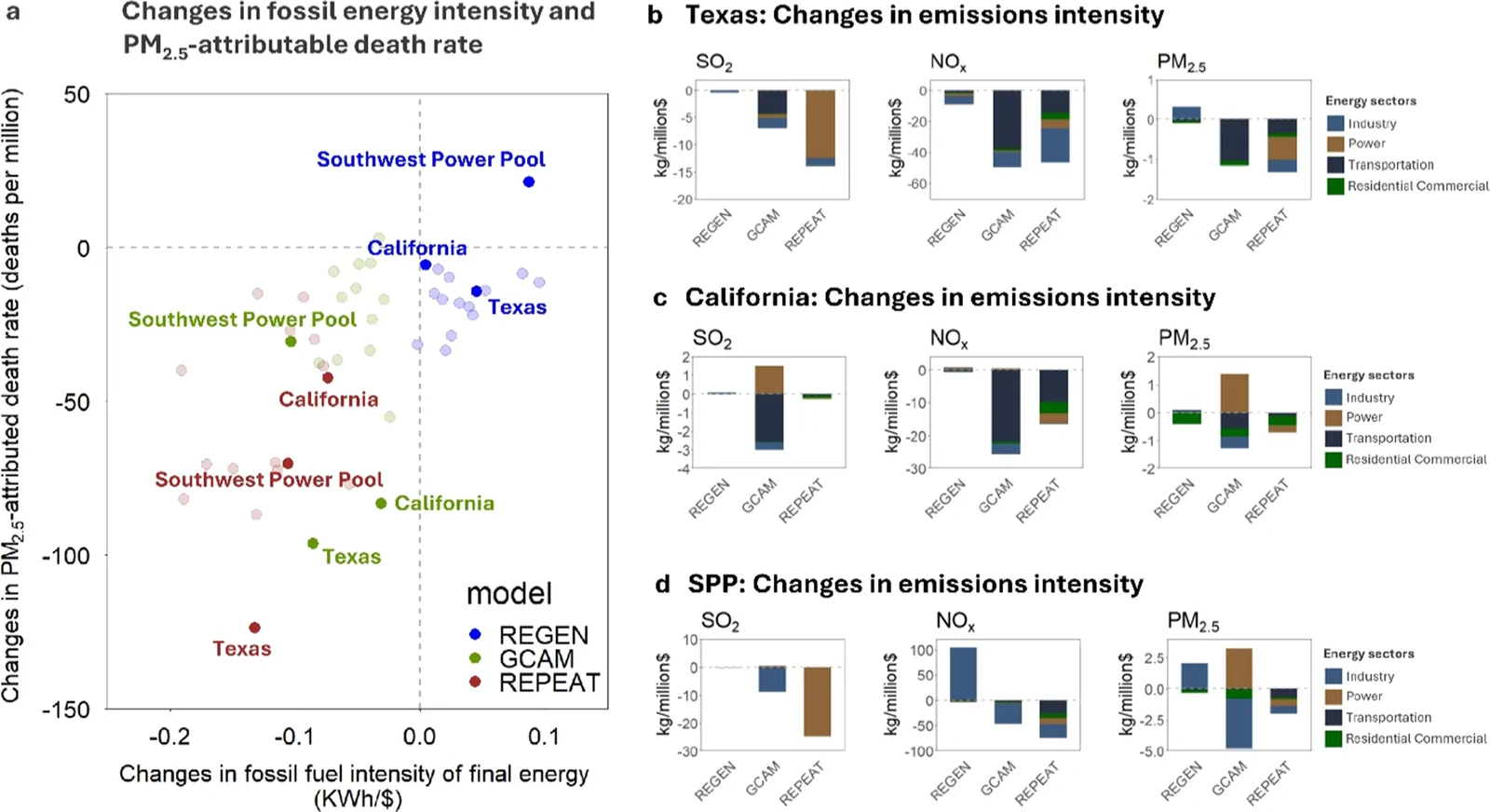
Changes in Net-Zero 2050 compared to Baseline 2050 for subnational regions. Panel a shows the relationship between changes in fossil fuel intensity (fossil-fuel final energy use divided by projected regional GDP) in final energy and changes in PM_2.5_-attributable death rates, with dots representing 14 subnational regions and colors representing the three models. Panels b–d show sectoral changes in energy-related emissions intensity (emissions divided by projected regional GDP) of SO_2_, NO_*x*_, and primary PM_2.5_ for three selected regions: Texas, California, and the Southwest Power Pool (SPP).

To further unpack the changes in the net-zero scenarios compared to the baseline scenarios, we show the relationship between the changes in fossil fuel intensity (fossil-fuel final energy use divided by projected regional GDP) and PM_2.5_-attributable death rates across 14 subnational regions (Figure 6). In GCAM-USA and REPEAT, a decrease in unabated fossil uses is often associated with a decrease in PM_2.5_-attributable death rates, though the strength of such a relationship varies across models and subnational regions, given the variations in local energy structures, emissions from non-energy sources, and meteorological conditions, as well as the size of exposed populations. In contrast, for most regions, the US-REGEN results show increases in unabated fossil use, despite reduced PM_2.5_-attributable death rates. This apparent divergence reflects that US-REGEN projects emission reductions in sectors with high population exposure, most notably transportation, rather than by changes in fossil fuel use in the power sector alone. Moreover, part of the remaining liquid fuel used in US-REGEN includes biofuels, such that aggregate liquid fuel metrics may overstate unabated fossil fuel combustion when interpreted without distinguishing fuel composition.

We then offer a deep dive into three subnational regions to demonstrate regional variations. For Texas, all models estimate net emission reductions in SO_2_ and NO_*x*_ in the Net-Zero scenarios compared to Baseline scenarios, with the greatest reductions found for REPEAT (Figure 6b). These patterns are driven by large reductions in fossil consumption in the state. For California, as a state where baseline emissions are already relatively low in several sectors, the changes in precursor emissions vary more across models: REPEAT projects substantial reductions in NO_*x*_ emissions from the transport sector as well as other sectors; GCAM-USA projects non-trivial increases in power-sector SO_2_ and PM_2.5_ emissions due to increased biomass combustion with CCS to achieve deep decarbonization, offsetting the benefits that could happen due to substantial reductions in NO_*x*_ emissions from the transport sector; and US-REGEN projects small changes in all types of pollutants (Figure 6c). Finally, for the Southwest Power Pool region (including Kansas, Nebraska, Oklahoma, and South Dakota), US-REGEN projects large increases in industrial NOx and PM_2.5_ emissions mainly from industrial sector gas use, and GCAM-USA projects a slight increase in power-sector SO_2_ emissions. In comparison, REPEAT projects greater reductions in SO_2_ and NO_*x*_ (Figure 6d). These state examples demonstrate how nationally defined net-zero pathways can translate into different state- and region-level emission outcomes through model-specific sectoral allocation of mitigation and residual combustion.

### 3.4 Robustness and Sensitivity Analyses

We conduct analyses to test whether the main findings are robust to assumptions that have been found to be important in prior studies focused on decarbonization and the health effects, including (a) timing,^21^ (b) net-zero target definition,^71^ (c) concentration–response functions,^60,64^ and (d) demographic projections.^72^ While these analyses do not sample the full uncertainty space, they offer insights into the core uncertainties that are most likely to influence our conclusions.

First, we consider 2030 as an intermediate year toward net-zero emissions. We find that the 2030 energy results show smaller cross-model differences in primary and final energy use than the 2050 results because the net-zero constraints are not yet fully binding and the energy systems have just started structural transformation (Figure S6). As a result, although the Net-Zero scenarios already reduce PM_2.5_-attributable mortality relative to the corresponding Baseline scenarios across models (Figure S7), the additional benefits are smaller than in 2050.

Second, using GCAM-USA, we compare a Net-Zero-CO_2_ case (only CO_2_ emissions) with a broader Net-Zero-GHG case (including both CO_2_ emissions and non-CO_2_ GHG emissions). We find that setting a net-zero GHG target slightly changes the energy pathway and residual GHG emissions, including somewhat higher primary and final energy use associated with additional mitigation that needs efforts from CDR (Figure S8). However, national PM_2.5_-attributable mortality remains similar across the two target definitions (Figure S8), suggesting that the specification of aggregate climate constraint does not strongly shape the PM_2.5_-attributable health conclusions.

Finally, we test uncertainties in the health impact assessment by using alternative relative risk functions and population projections. Using GEMM as an alternative relative-risk function reduces the absolute magnitude of estimated PM_2.5_-attributable mortality compared with the main Pope et al. function (Figure S9). Nevertheless, our key findings of the comparison of health benefits across models and between Baseline and Net-Zero scenarios remain consistent. We also test alternative population assumptions using SSP3 and SSP5 population distribution and aging patterns. These assumptions change national mortality estimates because county-level population distribution and age structure affect exposure and baseline mortality. However, because we rescale the SSP population projections to keep the national total population consistent with each energy model’s socioeconomic assumptions, the changes in national mortality magnitude are modest. More importantly, these modest changes do not affect our key conclusions when comparing across models and scenarios (Figure S10).

### 3.5 Discussion

Our multi-model analysis indicates that a Net-Zero transition will bring air quality and health benefits, but the scale and geographic distribution of these benefits are affected by the technology strategies being used to decarbonize the economy. Mitigating CO_2_ emissions generally lowers the level of health damage. Yet, the sectoral allocation of mitigation efforts and the technology strategies used to achieve CO_2_ reductions influence the associated changes in air pollutant emissions, thereby affecting the magnitude of the air quality improvements. Whether the pollution reductions occur in densely populated areas further determines the extent of population exposure and the resulting health impact.

Importantly, our results confirm a widely acknowledged insight that reducing fossil combustion is the core mechanism to maximize the health benefits.^2,15^ We further highlight that reducing fossil fuel use in some sectors (e.g., transportation and industry) brings more air-quality and health benefits than other sectors. This is especially important in net-zero pathways, where residual combustion may shift from the power sector toward harder-to-abate sectors, and where CDR strategies can alter the amount and location of remaining combustions.^16^ This finding is consistent with prior evidence that PM_2.5_ damages are highly source- and location-specific,^73^ and with previous U.S. deep decarbonization studies showing that air-quality and health co-benefits vary across technology pathways, sectors, and regions.^6,23,41^

Our analysis also highlights the importance of coordinating energy and non-energy activities to achieve both decarbonization and air quality goals. Land use and the agricultural sector act both as carbon sinks and as sources of some greenhouse gas emissions, such as methane.^74^ They also become increasingly important sources of air pollutant emissions, such as primary PM_2.5_ emissions from wildfires and NH_3_ emissions from agricultural activities.^74^ While the energy sector remains central to decarbonization, it is important to also consider these non-energy sectors, not only due to their own emissions but also because of their interactions with the energy system. For example, bioenergy with CCS is a key CDR technology to achieve decarbonization, but it has important implications for land use and management.^75,76^

We highlight the methodological limitations of the current study, which point to new directions for future research. Given our core focus on energy system models, we see three core areas for improvement. First, the net-zero scenarios here are developed based on the least-cost approach to reaching national net-zero targets, e.g., through an economy-wide carbon price or emissions cap. They do not include explicit state- and local-level policies, despite growing evidence that bottom-up climate action may be essential for advancing decarbonization in the United States given the current federal policy environment.^36,46^ To strengthen the policy relevance of future model comparison exercises, greater emphasis should be placed on modeling real-world energy transition policies and actions by examining a broader set of policy instruments^22^ (such as mandates, subsidies, and carbon markets) and accounting for how these policies evolve over time^77^ (such as policy sequencing and changes in policy ambition). Although individual modeling studies have increasingly assessed the air quality, health, and climate effects of specific policy choices and portfolios (see examples using GCAM-USA,^22^ US-REGEN,^41^ and REPEAT^42^), a coordinated intercomparison of these policy-focused analyses will help generate robust and decision-relevant insights.

Second, another aspect of policy relevance is the treatment of policy actions in countries beyond the U.S. Our analysis does not explicitly consider how rest-of-world policies affect U.S. PM_2.5_ exposure. Although GCAM-USA is embedded in a global modeling framework, our InMAP simulations focus on emissions within the continental U.S. This scope is appropriate for isolating the effects of U.S. energy-system pathways, but it omits intercontinental transport of air pollution^78^ and the effects of global climate on regional meteorology and therefore air pollution.^79^ Future work could link global mitigation scenarios with regional air quality models to consider cross-boundary pollution transport and climate-driven changes in wildfire, dust, biogenic emissions, and meteorology.

Furthermore, we focus only on air quality and health effects but do not offer a comprehensive cross-model comparison of the overall macroeconomic impacts of decarbonization. In our study, cost estimates are not directly comparable across the three models because the models consider and track different aspects of costs. Therefore, rather than interpreting one model as more or less costly than another based on non-harmonized cost outputs, we report the 2050 economy-wide carbon price (ranging from 194 to 212 in 2012 $ per ton CO_2_) as a limited but more comparable indicator of mitigation stringency. Yet, economic and wellbeing outcomes are highly relevant for comparing net-zero pathways, and recent assessments emphasize that decarbonization policies can have broad macroeconomic, affordability, and distributional implications.^80^ This leads to an important direction for future research.

There are also areas for improvement in our downstream emissions, air quality, and health assessments. For emission downscaling, in this study, we use the historical patterns based on NEI 2017 to downscale the emissions. While this approach has been widely applied in the modeling of future scenarios,^22,81,82^ it implicitly assumes that sectoral emission hotspots retain their present-day spatial distribution. Yet, in practice, decarbonization efforts are likely to change those spatial patterns by altering the geographic locations of retiring fossil fuel infrastructure and emerging emission sources, such as biomass energy facilities.^16,83^ Because air pollution and its health consequences are inherently local, future studies should place greater emphasis on the siting and retirement decisions of infrastructure as well as the implementation of local pollution control strategies. For air quality and health modeling, annual-average, population-weighted PM_2.5_ is used for the primary analysis of this study because our health impact assessment focuses on mortality associated with long-term exposure. Although long-term PM_2.5_ exposure contributes a substantially larger share of the air pollution mortality burden than others,^84^ this approach does not capture short-term health impacts or the health impacts of other pollutants such as ozone.^79,85^ Future research could address these limitations by using full chemical transport models (CTMs), incorporating additional pollutants (such as ozone), and short-term exposure-response relationships in the modeling framework. In addition, our study adopts one particular projection of socio-demographic patterns in the main analysis, such as aging and migration. However, studies have found that these fine-scale dynamics are crucial in determining the distribution of the impacts and the outcomes on exposure and health disparities.^86^

Finally, the study focuses on several important sources of uncertainty but still does not cover the full uncertainty space. Through our model comparison efforts, we consider structural differences among models. We have also considered a small but carefully selected set of factors for sensitivity analysis, such as alternative assumptions for emission targets, relative risk functions, and projections for sociodemographic changes and non-energy emissions. Future analyses should incorporate additional sources of uncertainty that have been found to be potentially important in the literature, such as changes in meteorological conditions^79^ and variations in technology innovation and adoption.^87^ Future work should also systematically evaluate the magnitude and relative importance of these uncertainties as well as how they propagate through the causal chain. Such efforts will likely require advanced uncertainty analysis methods, such as large-scale scenario ensembles or modeling to generate alternative (MGA) approaches to sample uncertainties,^88^ combined with scenario discovery methods to identify influential uncertainties and their interactions.^89^

## Supplementary Material



## Notes

Additional information regarding the data used as inputs for this study, including Tables S1–S6 and Figures S1–S10 (PDF)

## Notes

Code and Data availability: All data and code used to analyze the results and plot the figures are available from 10.5281/zenodo.22306874.
