# Supplementary material for: A Multi-Model Assessment of the Air Quality and Health Impacts of U.S. Energy Decarbonization
Source: Environ Sci Technol. 2026 Sep 17;60(38):27063–78. doi: 10.1021/acs.est.6c02914 (PMC13629629; doi:10.1021/acs.est.6c02914)
Supplement: Supplementary file 1 [file es6c02914_si_001.pdf]

Supporting Information for "A Multi-Model Assessment of the Air Quality and Health Impacts of US Energy Decarbonization"

Jinyu Shiwang[1,2,3], Wei Peng[1,2*], John Bistline[4,5], Jamil Farbes[6], Xinyuan Huang[2], Gokul Iyer[7], Jesse D. Jenkins[2,8], Eladio Knipping[9], Nicholas Mailloux[10], Erin Mayfield[11], Aranya Venkatesh[9], Alicia Zhao[7], Qianru Zhu[9]

[1] School of Public and International Affairs, Princeton University, Princeton, NJ 08544, USA

[2] Andlinger Center for Energy and the Environment, Princeton University, Princeton, NJ 08544, USA

[3] Department of Civil and Environmental Engineering, The Pennsylvania State University, University Park, PA 16802, USA

[4] Watershed, San Francisco, CA 94103, USA

[5] Massachusetts Institute of Technology, Cambridge, MA 02139, USA

[6] Evolved Energy Research, San Diego, CA 92101, USA

[7] Center for Global Sustainability, School of Public Policy, University of Maryland, College Park, MD 20742, USA

[8] Department of Mechanical and Aerospace Engineering, Princeton University, Princeton, NJ 08544, USA

[9] Electric Power Research Institute (EPRI), Palo Alto, CA 94304, USA

[10] Center for Sustainability and the Global Environment, Nelson Institute for Environmental Studies, University of Wisconsin–Madison, Madison, WI 53726, USA

[11] Thayer School of Engineering, Dartmouth College, Hanover, NH 03755, USA

* Corresponding author: weipeng@princeton.edu

This file contains 29 pages, 6 tables, and 10 figures.

This supplementary information includes the following information:

1. Additional details on models and methods

1.1 Detailed comparison about model structures

Table S1. Model structure comparison across three energy models

1.2 Detailed sector mapping for air pollutant emissions

Table S2. Details of the sector mapping between model-specific sectors and four aggregated categories

Table S3. Details of the sector mapping between NEI 2017 sectors and aggregated categories

1.3 Model inputs and outputs

Table S4. National-total non-energy emissions for the sensitivity runs

Table S5. National Population projection across three energy models

Table S6. National GDP projection across three energy models

2. Additional results for the main scenarios and sensitivity runs

2.1 Comparison across model base-years results and 2017

Figure S1. National energy patterns in 2017 and model base-year

Figure S2. National health results in 2017 and model base-year

2.2 Additional results for the main scenarios

Figure S3. National absolute health results

Figure S4. County-level changes in $PM_{2.5}$ concentrations and $PM_{2.5}$-attributable death rates.

Figure S5. Air pollutant emissions by sector in Florida

2.3 Comparisons across models in 2030

Figure S6. National energy patterns in 2030

Figure S7. National health results in 2030

2.4 Comparison between Net-Zero-CO_2 and Net-Zero-GHG scenarios

Figure S8. National health results between the Net-Zero-CO_2 scenario and the Net-Zero-GHG scenario in GCAM-USA

2.5 Sensitivity analysis in health impact assessment

Figure S9. National health results using alternative relative risk (RR) function

Figure S10. National health results using alternative SSP assumptions for population

1. Additional details on models and methods

1.1 Detailed comparison about model structures

Table S1. Model structure comparison across three energy models (The GCAM-USA model descriptions are adapted from Luo et al., 2026[1])

	GCAM-USA	US-REGEN	REPEAT
**Purpose of the model**	(1) Integrated approach: The model aims to simulate the interactions between five systems – socioeconomic, energy, climate, water, and land.<br>(2) Impacts: The model has detailed representation of the carbon intensity of energy technologies and land options to evaluate the impacts on GHG emissions and the climate system.	(1) Integrated approach: Links a detailed electric-sector capacity-expansion/dispatch and fuels-supply model with technologically rich end-use demand modules (buildings, transport, industry).<br>(2) Impacts: Resolves regional resource and policy heterogeneity and transmission, enabling analysis of U.S. energy system transition pathways, policy design, technology and infrastructure deployment, emissions, and system costs.	(1) Integrated approach: The model employs a suite of geospatial planning and energy-demand scenario-based analysis tools coupled with a detailed macro-energy system optimization model capable of rapidly evaluating policy and regulatory proposals at politically-relevant spatial resolutions (e.g., state, county, and sometimes finer resolutions).<br>(2) Impacts: It provides a detailed look at the United States' evolving policy environment and the country's progress on the road to net-zero greenhouse gas emissions, supporting analysis around technology and infrastructure deployment as well as emissions and cost impacts.

**Structure of the model:** Degree of endogenization	(1) Key exogenous inputs: Variables: Socioeconomic factors (e.g., population and labor productivity), energy technology costs, resource supply curves, emission targets, etc; Parameter values: Demand elasticities, specification of choice functions (e.g., shareweights and logit exponents), emission factors, etc.<br><br>(2) Key endogenous processes: Energy demand is estimated endogenously based on socioeconomic drivers and relevant elasticities, both of which are specified as exogenous assumptions. The choices of energy supply technologies are modeled endogenously based on choice functions that consider the costs of competing technologies as well as the logit functions and technology shareweights that determine the relative importance of costs (or relative costs) and non-cost factors; the costs, logit functional forms, and shareweights are based on exogenous assumptions; resource availability constraints are further imposed for relevant technologies (e.g., wind, solar, biomass). Trade of energy commodities	(1) Key exogenous inputs: Socioeconomic assumptions and service demand; technology cost/performance; resource supply curves (renewables including biomass, CO_2 transport and storage, natural climate solutions); select fuel prices; federal and state policies including emissions (prices or caps) and clean energy targets, emissions factors.<br><br>(2) Key endogenous processes: Capacity expansion and chronological dispatch; transmission builds; fuel supply and infrastructure; energy storage investment and operations; technology/fuel switching in end uses subject to costs, constraints, and policies; hourly electric load shapes.<br><br>(3) Key outputs: Electric sector generation mix, capacity, and transmission; fuel production and trade; end-use energy by sector/fuel/technology; emissions (CO_2 and criteria pollutants); system costs and prices.	(1) Key exogenous inputs: Projected service demand; technology cost/performance; resource potential and quality (renewables, biomass, CO_2 transport and storage); existing energy infrastructure and equipment stocks; fuel prices; federal and state policies, including emissions (prices or caps) and clean energy targets.<br><br>(2) Key endogenous processes: Stock rollover and projections translating service demand into final energy demand projection and associated hourly electric load shapes. Capacity expansion for the full energy system subject to costs, constraints, and policies, including transmission builds; fuel supply and infrastructure; electric generation deployment; energy storage investment and operations.<br><br>(3) Key outputs: end-use technology deployment and final energy demand, electric sector generation mix, capacity, and transmission; fuel production; emissions (CO_2 and criteria pollutants);

	across states or regions, such as electricity and bioliquids, is modeled endogenously by combining locally produced and imported commodities in the supply curves. The carbon price is computed endogenously by the model as the marginal abatement costs to reach a specified emission target; here emission targets are implemented as an exogenously specified constraint, and the marginal mitigation cost curves are largely exogenously specified (e.g., based on the emission intensity and maximum possible mitigation potential of a wide range of technologies).<br><br>(3) Key outputs: Detailed energy demand and supply by technology choice and state/region, emissions by fuel, technology, and state/region, Carbon price level to meet the emission target, Other market prices, e.g., electricity, passenger vehicle etc.		system costs and prices.

**Structure of the model:** Description of non-energy sectors	Land and water systems are modeled at the subnational scale for the US, whereas the global climate system is modeled in the global GCAM-USA model. For land, the model uses a nesting strategy to model economic competition between roughly 10 broad land types. For water, it includes three types of water supply (renewable, non-renewable and desalinated) and a range of water-consuming technologies across electricity, residential, industrial and agricultural sectors.	Primarily energy-focused; land/agriculture modeled only as they pertain to bioenergy feedstocks/fuel supply and selected natural climate solutions; broader land/water systems are exogenous.	Energy-focused; agricultural and land-use emissions are estimated outside of the model and incorporated into REPEAT results packages.
**Structure of the model:** Description of end-uses	The model includes detailed representation of the transportation sector (divided into four types of final demand covering 60 technologies) and building sectors (disaggregated into residential and commercial sectors; models floor space and heating, cooling and other services), with some representation of the industry sector (including eight industrial sectors/processes).	The model includes structural details across several dimensions relevant for fuel and technology choice, such as building size, type, and vintage, climate zone and location, and vehicle ownership and driving intensity. US-REGEN represents detailed technology choice and service demands in buildings, transport, and selected industry uses.	Detailed representation of energy end-use technologies across buildings, transportation, and industry, with more than 60 energy system subsectors, incorporating dimensions for fuel, building type, technology choice, and location/climate zone. The model also includes evaluation of candidate sites for wind and solar development, thermal power plant sitting and repowering, transmission expansion, and changes in energy sector employment.

**Structure of the model:** Description of supply technologies	The model includes a range of technologies in the power sector (Hydro, Solar (PV, thermal, rooftop PV), Geothermal, Wind, Biomass, Fossil, Fossil with CCS, Biomass with CCS, coal, gas, refined liquids-fired power plants, combined cycle with or without CCS), transportation (Aviation, Rail, Shipping, and road transportation (trucks, buses, cars), each having BEV, FCEV, ICEV and hybrid technologies), and building sectors, including commercial and residential cooking (range, oven, dishwasher, refrigerator), heating (furnace, air conditioning, heat pump), cooling, ventilation, lighting, office equipment, clothes washer & dryer, water heater, using different fuels (electricity, gas, biomass, hydrogen) with different levels of energy efficiency.	See the detailed US-REGEN model documentation for representations of primary energy conversion, delivery, and storage options: https://us-US-REGEN-docs.epri.com/v2025/	Power sector technologies include: hydro, solar (utility-scale PV, rooftop PV), geothermal, wind (onshore and offshore), biomass, gas, gas with CCS, biomass with CCS. Other supply technologies include: various forms of hydrogen production from fossil, biomass or electric inputs; Fischer-Trosch fuel synthesis; various biomass-derived fuel pathways; electric and hydrogen boilers; various steel and cement production pathways for lower emissions.
**Geographical coverage**	Global and national: State-level representation of socioeconomics, energy supply and demand within the US, along with 31 geopolitical regions outside the US that have market linkages with the US.	By default, the model uses 16 sub-regions, each an aggregation of states of US, but the model can be configured with any aggregation of the lower 48 states or in some cases sub-state areas.	Geographical coverage is highly extensible, with US analyses typically done with 27 sub-regions. Coverage can be modified for sub-national studies or national studies outside the US.

**Sectoral coverage**	Economy-wide, including energy, land, water, land systems	Economy-wide energy (electric supply and demand; buildings; transport; industry) with linked fuels supply/logistics.	Economy-wide energy (electric supply and demand; buildings; transport; industry) with linked fuels supply.
**The time horizon**	Medium to long term: This study focuses on 2021-2050, whereas the model can run from 1975-2100	Medium to long term: 2015 through 2050	Medium to long term: 2022 through 2050
**Time step**	5-year time-steps	5-year time-steps	2022, 2024, 2026, 2028, 2030, 2032, 2035, 2040, 2050
**Renewable Technology Inclusion**	Hydro, Solar (PV, thermal, rooftop PV), Geothermal, Wind, Biomass, Fossil, Fossil with CCS, Biomass with CCS.	Onshore/offshore wind, utility-scale and rooftop PV, CSP, hydro, geothermal, biomass (with and without CCS).	Onshore/offshore wind, utility-scale and rooftop PV, hydro, geothermal, biomass (with and without CCS).
**Storage Technology Inclusion**	Battery energy storage in the electricity sector; Hydrogen storage such as underground storage and other storage methods such as high-pressure tanks and cryogenic tanks	Li-ion and other batteries, pumped hydro, thermal storage (where applicable), hydrogen storage and associated delivery.	Li-ion and other batteries, pumped hydro, thermal storage, hydrogen storage and associated delivery.
**Demand Characteristic Inclusion: Transport**	Aviation, Rail, Shipping, and road transportation (trucks, buses, cars), each having BEV, FCEV, ICEV and hybrid technologies.	Light-Duty Vehicles, On-Road Fleet and Non-Road Vehicles (including rail, aviation, shipping, non-manufacturing energy use such as in agriculture, construction and mining)	Light-Duty Vehicles, Medium- and Heavy-Duty Vehicles and Non-Road Vehicles (including rail, aviation, shipping, non-manufacturing energy use such as in agriculture, construction and mining)

**Demand Characteristic Inclusion: Building**	Commercial and residential sectors, including different technologies for heating, cooling, cooking, lighting etc.	Residential & commercial end uses (space heating/cooling, water heating, cooking, dryers, lighting, data centers/other) that allows equipment/device/fuel choice. The model includes technology details for building stock with different vintages and in different climate zones.	Residential & commercial end uses (space heating/cooling, water heating, cooking, dryers, lighting, data centers/other) that allows equipment/device/fuel choice. The model includes technology details for building stock with different vintages and in different climate zones.
**Demand Characteristic Inclusion: Industry**	Cement, fertilizer, CO_2 removal, other industrial energy use, refinery	The industrial sector is modeled in detail for some sectors and technologies, including iron & steel, cement, and steam demand from boilers across all manufacturing sectors, while other industrial energy use is modeled with a more aggregate representation.	The industrial sector is modeled in detail for some sectors and technologies, including iron & steel, cement, and steam demand from boilers across all manufacturing sectors, while other industrial energy use is modeled with a more aggregate representation.
**Cost Inclusion**	Fuel price, capital cost, operational & maintenance costs, interest rates, policy costs (subsidy/carbon price)	Capital, fixed/variable O&M, fuel prices, transmission costs, CO_2 transport & storage costs, and policy instruments (e.g., taxes, credits); can include non-energy externalities (air-quality) in companion analyses.	Capital, fixed/variable O&M, fuel prices, transmission costs, CO_2 transport & storage costs, and policy instruments (e.g., taxes, credits) and capability to price non-energy externalities.
**The Analytical Approach**	Market equilibrium model	Least-cost system optimization with integrated fuels supply; co-optimizes capacity, dispatch, storage, and transmission under policy/resource	Scenario-approach for energy demand coupled with a least-cost system optimization for energy supply,

		constraints; end-use models for transport and buildings represented as lagged logit choice simulation.	integrating transmission and fuels supply.
**The Underlying Methodology**	- The representative agents use information on prices and make decisions about the allocation of resources.<br>- GCAM-USA solves for a set of market prices so that supplies and demands are balanced in all these markets across the model<br>- market equilibrium is assumed to take place in each one of these markets (partial equilibrium), and not in the entire economy across all markets (general equilibrium).	US-REGEN links a detailed electric sector capacity planning and fuels supply model with representations of demand in buildings, transport, and industry. The model jointly optimizes regional investments in electricity generation and energy storage, system dispatch, transmission, and non-electric fuels supply, which includes high temporal resolution and chronology to better characterize the economic incentives of variable renewables, energy storage, and dispatchable generation.	The REPEAT Project employs a suite of geospatial planning and analysis tools coupled with detailed macro-energy system optimization models capable of rapidly evaluating policy and regulatory proposals at politically-relevant spatial resolutions (e.g., state, county, and sometimes finer resolutions).
**The Mathematical Approach**	Numerical methods, i.e., iterating on market prices until this equilibrium is reached	The electric-fuels and end-use models are solved iteratively to convergence, allowing analysis of policy impacts on the electric and fuels supply sectors considering electricity and fuels demand responses, and conversely allowing analysis of how economy-wide energy policies and technological improvements impact energy demands.	The energy supply model is solved iteratively to convergence, allowing analysis of policy impacts on the electric and fuels supply sectors.

**Data Requirements**	Quantitative	Quantitative	Quantitative

1.2 Detailed sector mapping for air pollutant emissions

Table S2. Details of the sector mapping between model-specific sectors and four aggregated categories

Categories	US-REGEN sectors	GCAM-USA sectors	REPEAT sectors
Industry	Bulk chemicals<br>Iron and steel (primary and secondary)<br>Paper pulp and wood<br>Food<br>Cement<br>Aluminum<br>Specialized Manufacturing<br>Other manufacturing<br>Construction<br>Mining	airCO2<br>biomass liquids<br>cement<br>coal to liquids<br>gas pipeline<br>gas processing<br>gas to liquids<br>H2 central production<br>H2 wholesale dispensing<br>industrial processes<br>N fertilizer<br>oil refining<br>other industrial energy use<br>other industrial feedstocks<br>process heat cement<br>process heat dac<br>regional biomass<br>regional biomassOil<br>regional corn for ethanol<br>coal<br>crude oil<br>natural gas	Fuel Comb - Industrial Boilers, ICEs - Biomass<br>Fuel Comb - Industrial Boilers, ICEs - Coal<br>Fuel Comb - Industrial Boilers, ICEs - Natural Gas<br>Fuel Comb - Industrial Boilers, ICEs - Oil<br>Fuel Comb - Industrial Boilers, ICEs - Other<br>Industrial Processes - Cement Manuf<br>Industrial Processes - Chemical Manuf<br>Industrial Processes - Coal Mining<br>Industrial Processes - Ferrous Metals<br>Industrial Processes - Mining<br>Industrial Processes - NEC<br>Industrial Processes - Non-ferrous Metals<br>Industrial Processes - Oil & Gas Production<br>Industrial Processes - Petroleum Refineries<br>Industrial Processes - Pulp & Paper<br>Industrial Processes - Storage and Transfer<br>Miscellaneous Non-Industrial NEC<br>Solvent - Consumer & Commercial Solvent Use<br>Solvent - Degreasing<br>Solvent - Dry Cleaning<br>Solvent - Graphic Arts<br>Solvent - Industrial Surface Coating & Solvent Use<br>Solvent - Non-Industrial Surface Coating
Power	US-REGEN uses separate sources	backup_electricity	Fuel Comb - Electric Generation - Coal

	for emissions in the power sector, and the power sector name in US-REGEN: elec	base load generation<br>csp_backup<br>intermediate generation<br>peak generation<br>subpeak generation	Fuel Comb - Electric Generation - Natural Gas<br>Fuel Comb - Electric Generation - Oil<br>Fuel Comb - Electric Generation - Other<br>Fuel Comb - Electric Generation - Biomass
Transportation	Light duty vehicle<br>Aviation<br>Freight rail<br>International shipping<br>Domestic shipping<br>Heavy-duty trucks<br>Other passenger (mainly bus)<br>Airports<br>Railroad support<br>Marine ports	trn_aviation_intl<br>trn_freight<br>trn_freight_road<br>trn_pass<br>trn_pass_road<br>trn_pass_road_LDV<br>trn_pass_road_LDV_4W<br>trn_shipping_intl	Mobile - Aircraft<br>Mobile - Commercial Marine Vessels<br>Mobile - Locomotives<br>Mobile - Non-Road Equipment - Diesel<br>Mobile - Non-Road Equipment - Gasoline<br>Mobile - Non-Road Equipment - Other<br>Mobile - On-Road Diesel Heavy Duty Vehicles<br>Mobile - On-Road Diesel Light Duty Vehicles<br>Mobile - On-Road non-Diesel Heavy Duty Vehicles<br>Mobile - On-Road non-Diesel Light Duty Vehicles
Residential Commercial	Residential space cooling<br>Residential space heating<br>Residential water heating<br>Residential cooking<br>Residential clothing dryers<br>Residential Lighting<br>Electronics<br>Appliances (including ventilation, miscellaneous uses)<br>Commercial space cooling<br>Commercial space heating<br>Commercial water heating	comm cooking<br>comm cooling<br>comm heating<br>comm hot water<br>comm other<br>district heat<br>resid clothes dryers<br>resid cooking<br>resid cooling<br>resid heating<br>resid hot water<br>resid other<br>urban processes	Bulk Gasoline Terminals<br>Commercial Cooking<br>Fuel Comb - Comm/Institutional - Biomass<br>Fuel Comb - Comm/Institutional - Coal<br>Fuel Comb - Comm/Institutional - Natural Gas<br>Fuel Comb - Comm/Institutional - Oil<br>Fuel Comb - Comm/Institutional - Other<br>Fuel Comb - Residential - Natural Gas<br>Fuel Comb - Residential - Oil<br>Fuel Comb - Residential - Other<br>Fuel Comb - Residential - Wood<br>Gas Stations<br>Waste Disposal

Table S3. Details of the sector mapping between NEI 2017 sectors and aggregated categories

Aggregated sectors	NEI sectors
Industry	ptnonipm, pt_oilgas, np_oilgas, nonroad
Power	ptegu
Transportation	onroad, onroad_ca_adj, airport, cmv_c1c2, cmv_c3, rail, airports
Residential commercial	nonpt, rwc
Fugitive dust	afdust
Agriculture	ag, ptagfire
Wildfire	ptfire

1.3 Model inputs and outputs

Table S4. National-total non-energy emissions for the sensitivity runs

Scenario	Fire $PM_{2.5}$ (Tg/yr)	Agriculture NH_3 (Tg/yr)	Fugitive dust $PM_{2.5}$ (Tg/yr)
Main scenario (NEI 2017)	1.46	3.13	0.81
High non-energy projection scenario	5.51	4.96	1.13

Table S5. National-total population in 2017 and in three energy models

2017	322,821,400
Model base-year	
2015 US-REGEN	319,701,500
2021 GCAM-USA	332,048,000
2022 REPEAT	333,000,000
Model projected	
2030 US-REGEN	357,352,800
2030 GCAM-USA	349,684,000
2030 REPEAT	347,000,000
2050 US-REGEN	396,112,900
2050 GCAM-USA	389,660,000
2050 REPEAT	376,000,000

Table S6. National-total GDP in three energy models

Model base-year	
2015 US-REGEN	16,549 (billion 2012 $)
2021 GCAM-USA	21,532 (billion 2012 $)
2022 REPEAT	20,303 (billion 2012 $)
Model projected	
2030 US-REGEN	23,289 (billion 2012 $)
2030 GCAM-USA	27,930 (billion 2012 $)
2030 REPEAT	24,555 (billion 2012 $)
2050 US-REGEN	34,365 (billion 2012 $)
2050 GCAM-USA	41,281 (billion 2012 $)
2050 REPEAT	36,652 (billion 2012 $)

2. Additional results for the main scenarios and sensitivity runs

2.1 Comparison across model base-years results and 2017

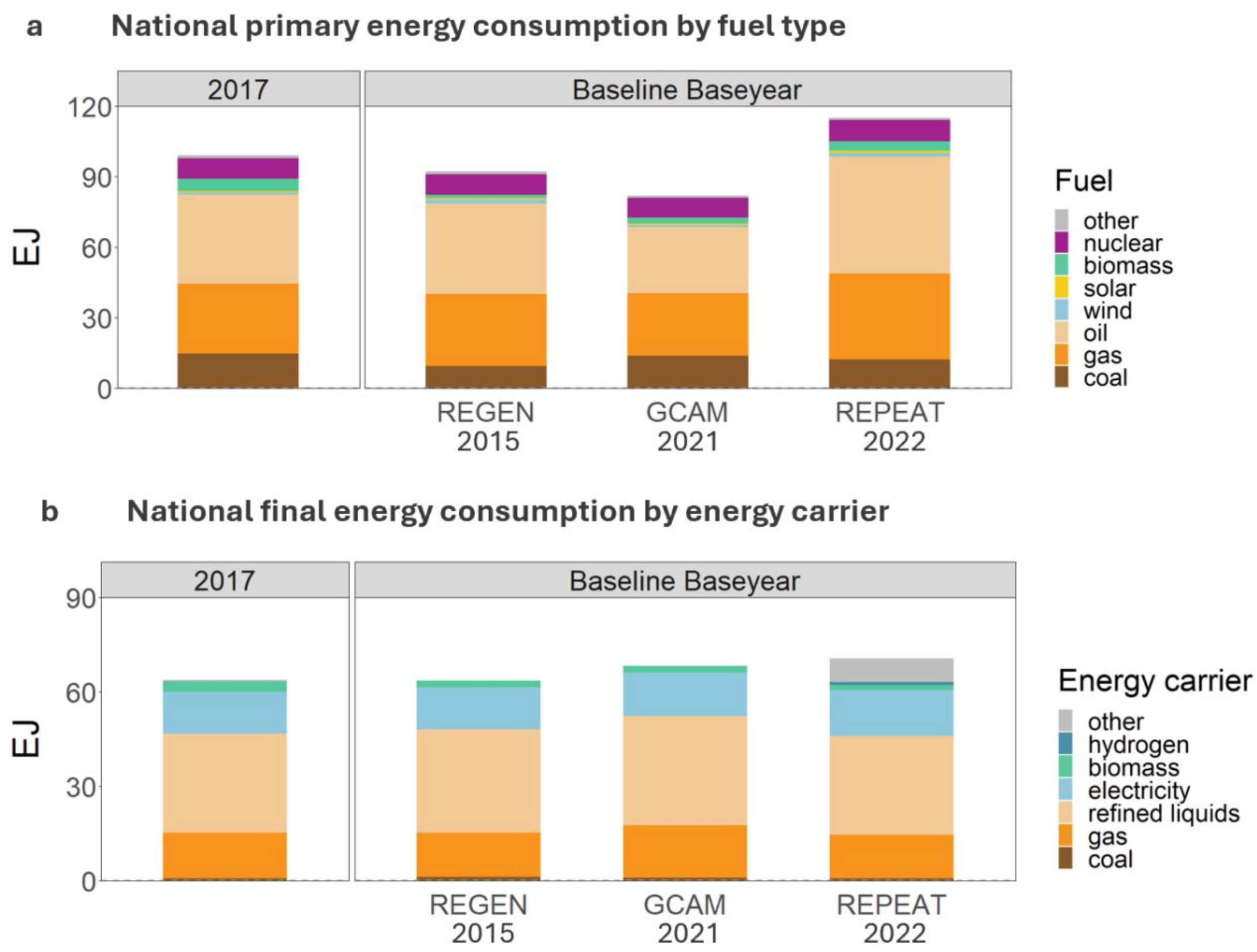

Figure S1. National energy patterns in 2017 and model base-year. Panels show national primary energy consumption by fuel type and final energy consumption by energy carrier for the historical 2017 benchmark and each model's native base-year scenario. Model base years are 2015 for US-REGEN, 2021 for GCAM-USA, and 2022 for REPEAT.

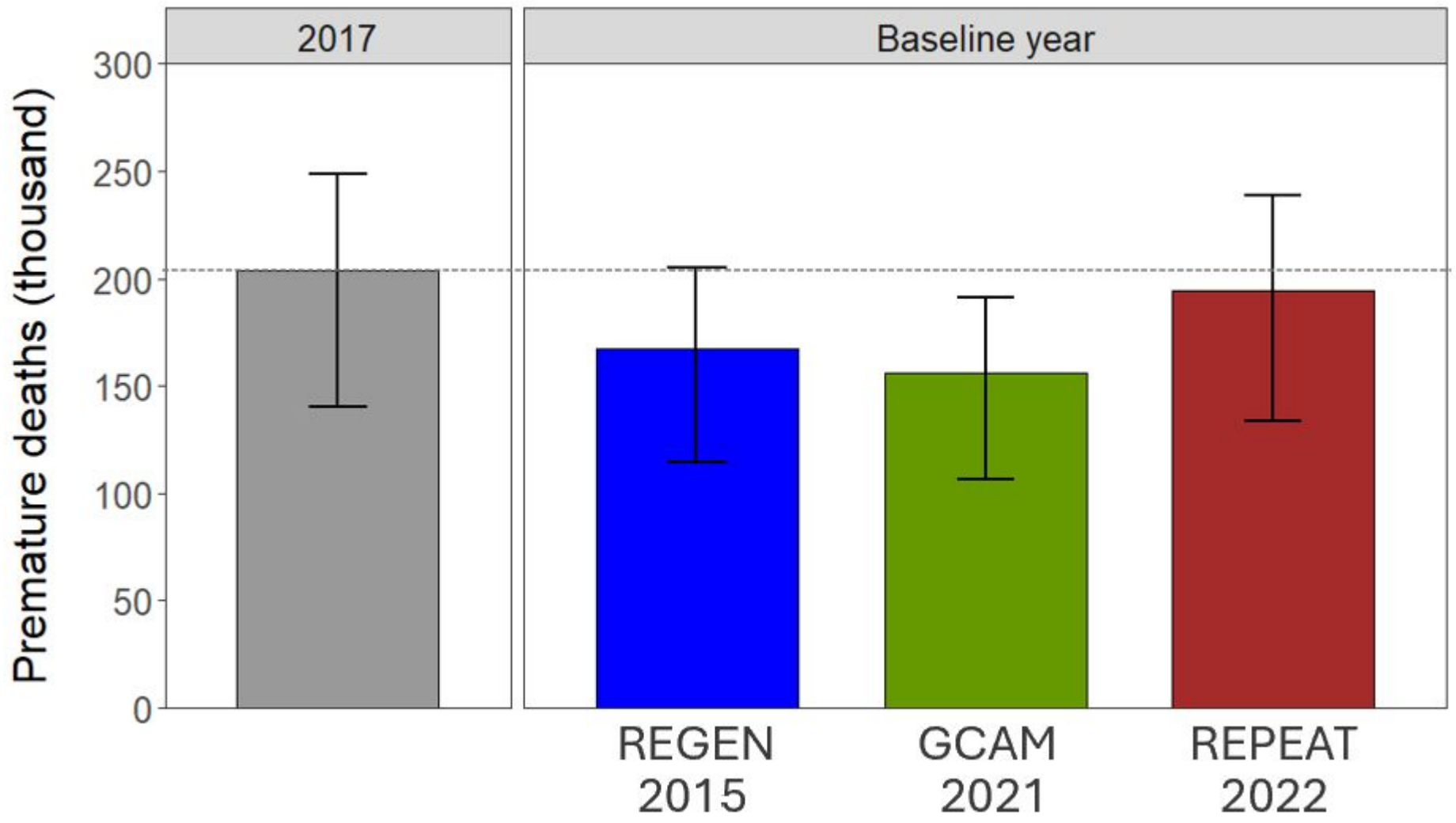

Figure S2. National health results in 2017 and model base-year. The 2017 bar represents the historical benchmark based on NEI2017 emissions. The model base-year bars show estimates using each model's native base-year energy emissions combined with the harmonized health-impact assessment workflow. Model base years are 2015 for US-REGEN, 2021 for GCAM-USA, and 2022 for REPEAT. Error bars indicate 95% CI in the relative risk function.

2.2 Additional results for the main scenarios

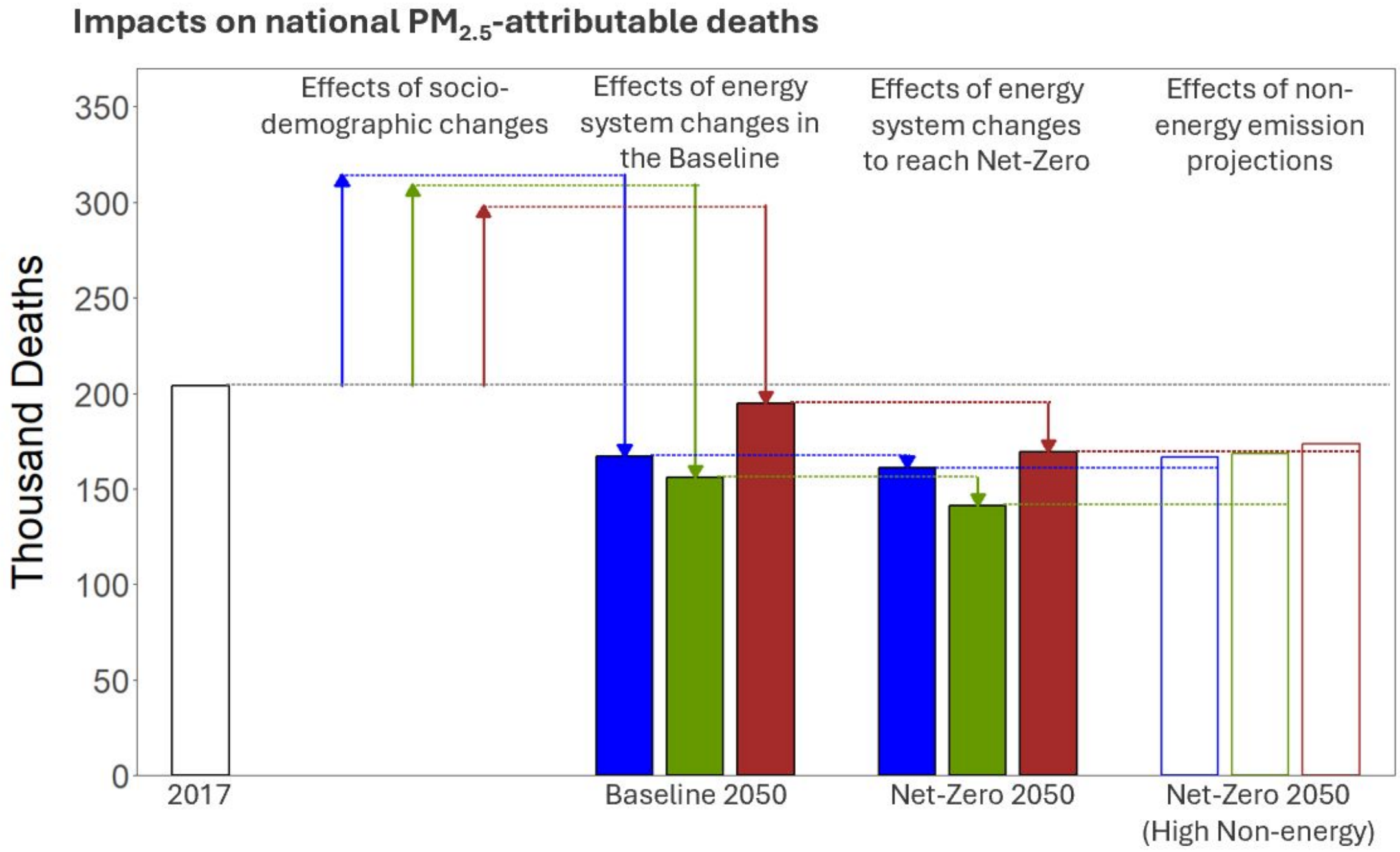

Figure S3. Effects of socioeconomic and technological factors on national health effects with absolute deaths. Panels show the relative contributions of socio-demographic, energy system changes, and non-energy factors to the differences in national $PM_{2.5}$-attributable deaths (thousand) between 2017, Baseline 2050, and Net-Zero 2050 scenarios. Bars represent model-specific estimates for 2017, and the 2050 scenarios, and arrows illustrate the effects of each factor, including the direction and the magnitude. The effects of non-energy emissions are quantified by applying high projected emissions from wildfire and agricultural activities from other datasets. The three colors represent the three models: US-REGEN (blue), GCAM-USA (green), and REPEAT (brown).

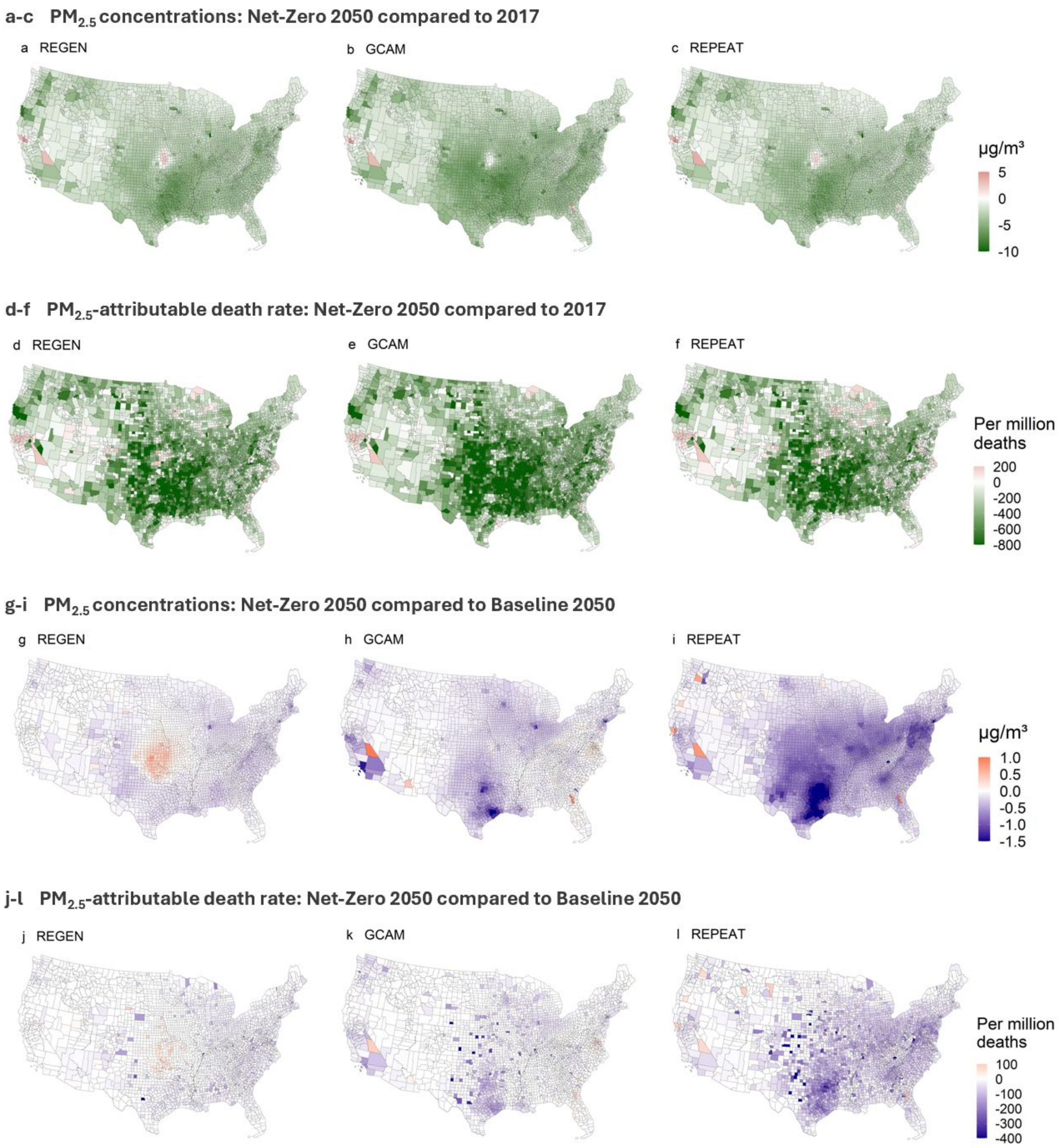

Figure S4. County-level changes in $PM_{2.5}$ concentrations and $PM_{2.5}$-attributable death rates. Panels show changes in annual average $PM_{2.5}$ concentrations (panels a-c, g-i) and $PM_{2.5}$-attributable death rates (panels d-f, j-l) across U.S. counties under Net-Zero 2050 scenarios relative to 2017 and Baseline 2050 for the US-REGEN, GCAM-USA, and REPEAT models, respectively. Panels a-f show reductions in $PM_{2.5}$ concentrations and associated mortality between Net-Zero 2050 and 2017, while panels g-l show the additional improvements achieved when transitioning from Baseline 2050 to Net-Zero 2050. Deeper colors indicate larger benefits, with green representing benefits relative to 2017 and purple representing additional benefits relative to Baseline 2050. Localized county-level hotspots reflect the interaction between model-projected sectoral emissions changes and static 2017 spatial downscaling factors

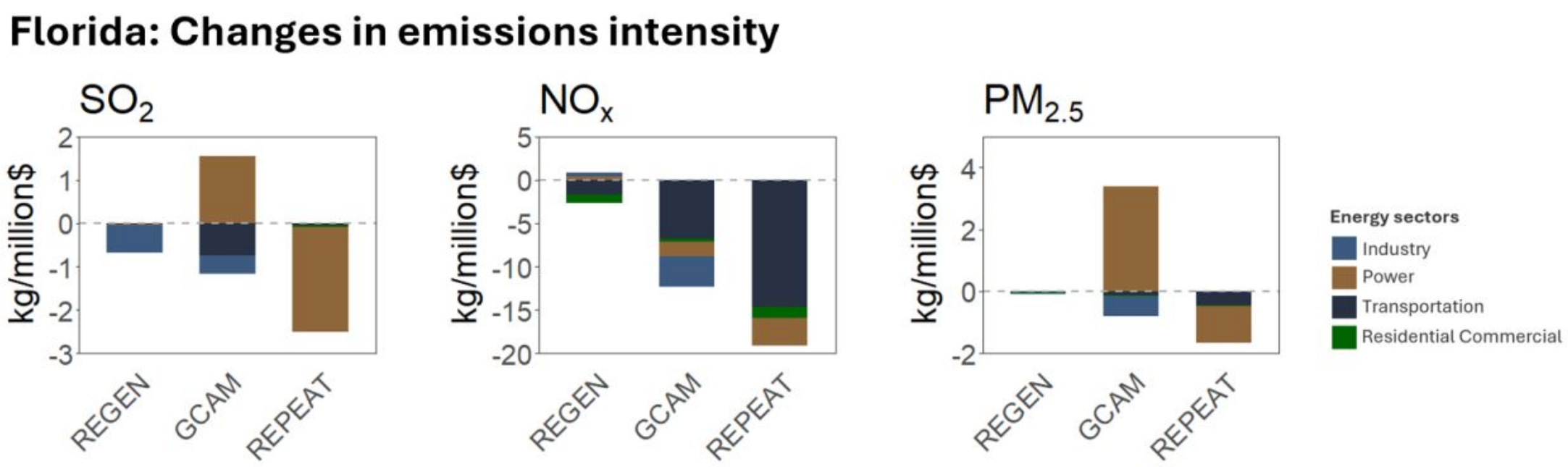

Figure S5. Sectoral changes in energy-related emissions intensity of SO_2, NO_x, and primary $PM_{2.5}$ for Florida between Net-Zero 2050 and Baseline 2050.

2.3 Comparisons across models in 2030

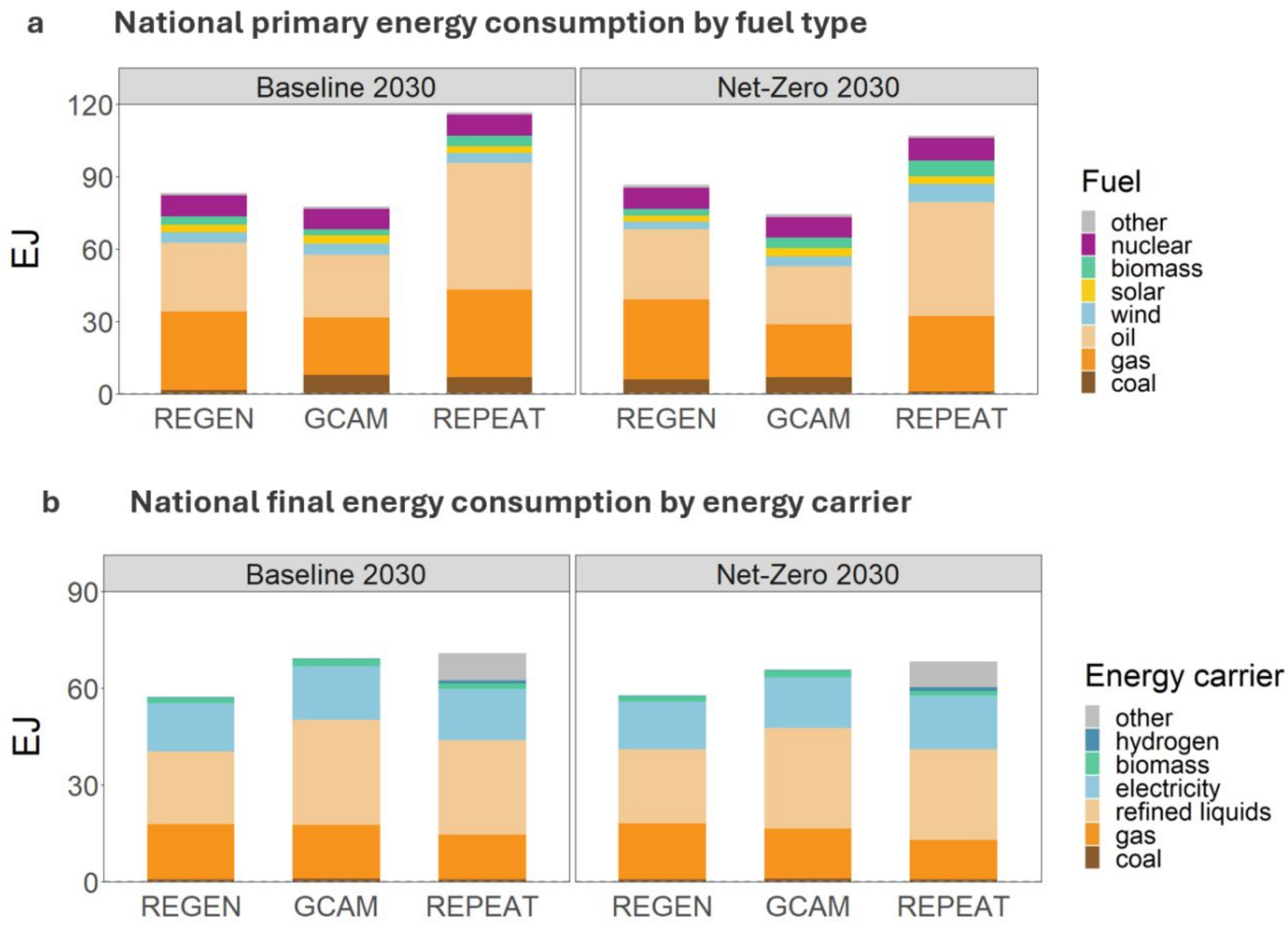

Figure S6. National energy patterns in 2030. Panels show national primary energy consumption by fuel type and final energy consumption by energy carrier under Baseline 2030 and Net-Zero 2030 scenarios for US-REGEN, GCAM-USA, and REPEAT.

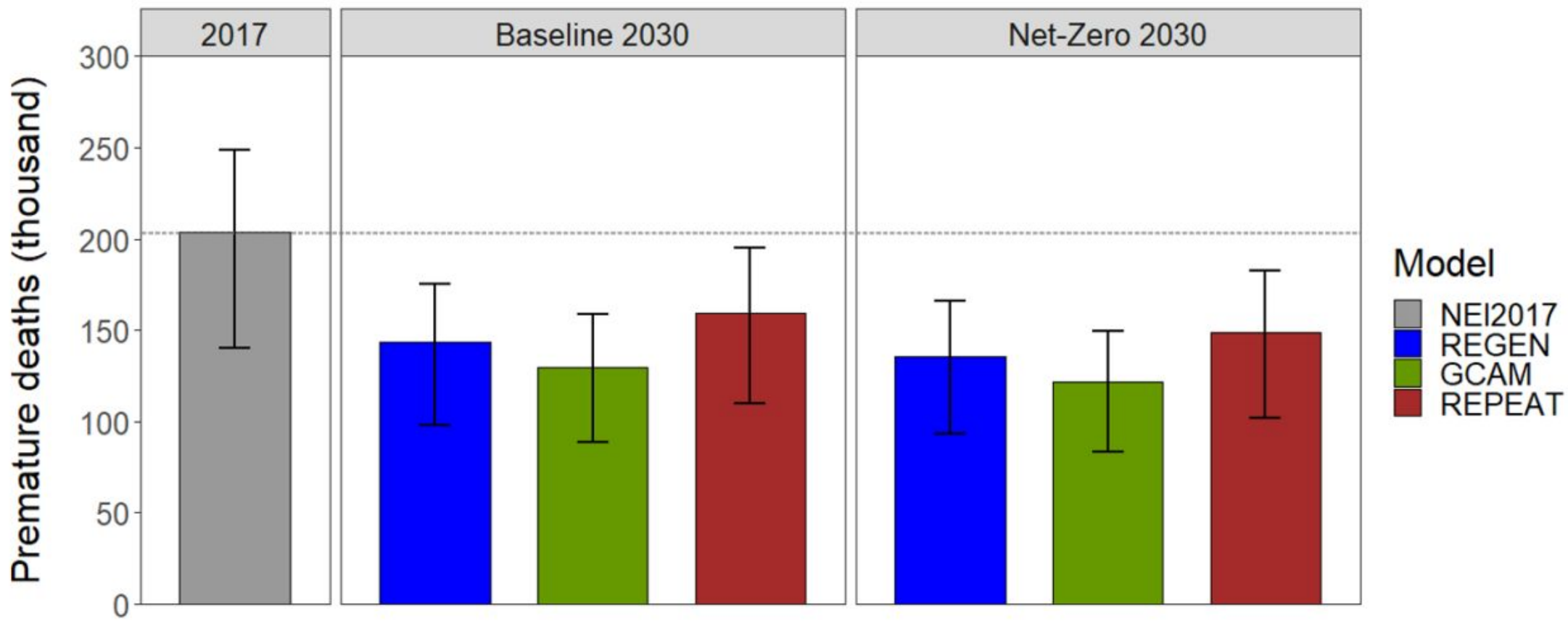

Figure S7. National health results in 2030. Bars show national PM2.5-attributable deaths in 2017, Baseline 2030, and Net-Zero 2030. Error bars indicate 95% CI from the relative risk function. The dashed horizontal line indicates the 2017 estimate.

2.4 Comparison between Net-Zero-CO2 and Net-Zera-GHG scenarios

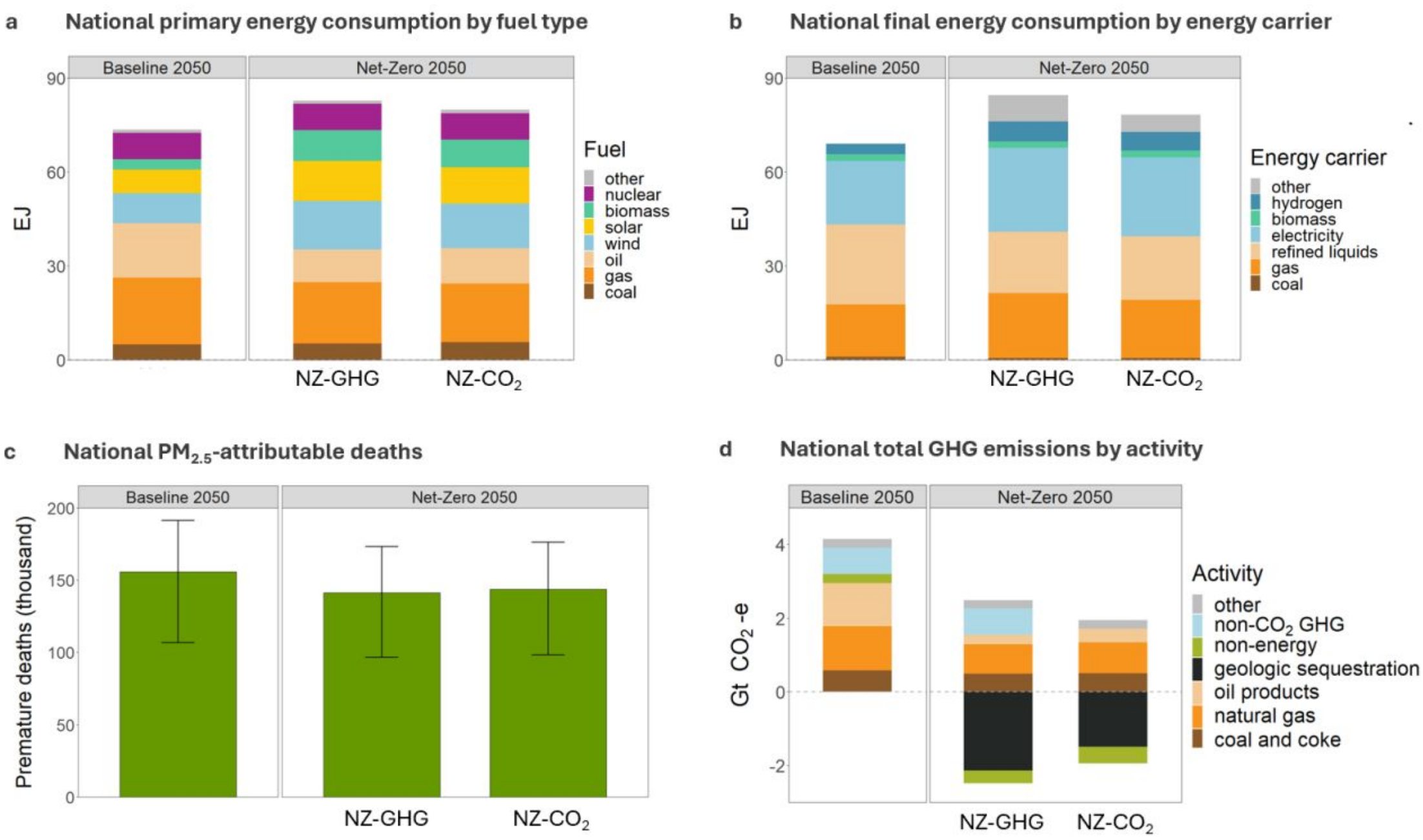

Figure S8. Comparison between Net-Zero-CO_2 and Net-Zero-GHG scenarios in GCAM-USA. Panels show national primary energy consumption by fuel type, final energy consumption by energy carrier, $PM_{2.5}$-attributable deaths, and total GHG emissions by activity under the Baseline 2050, Net-Zero-GHG, and Net-Zero-CO_2 scenarios.

2.5 Sensitivity analysis in health impact assessment

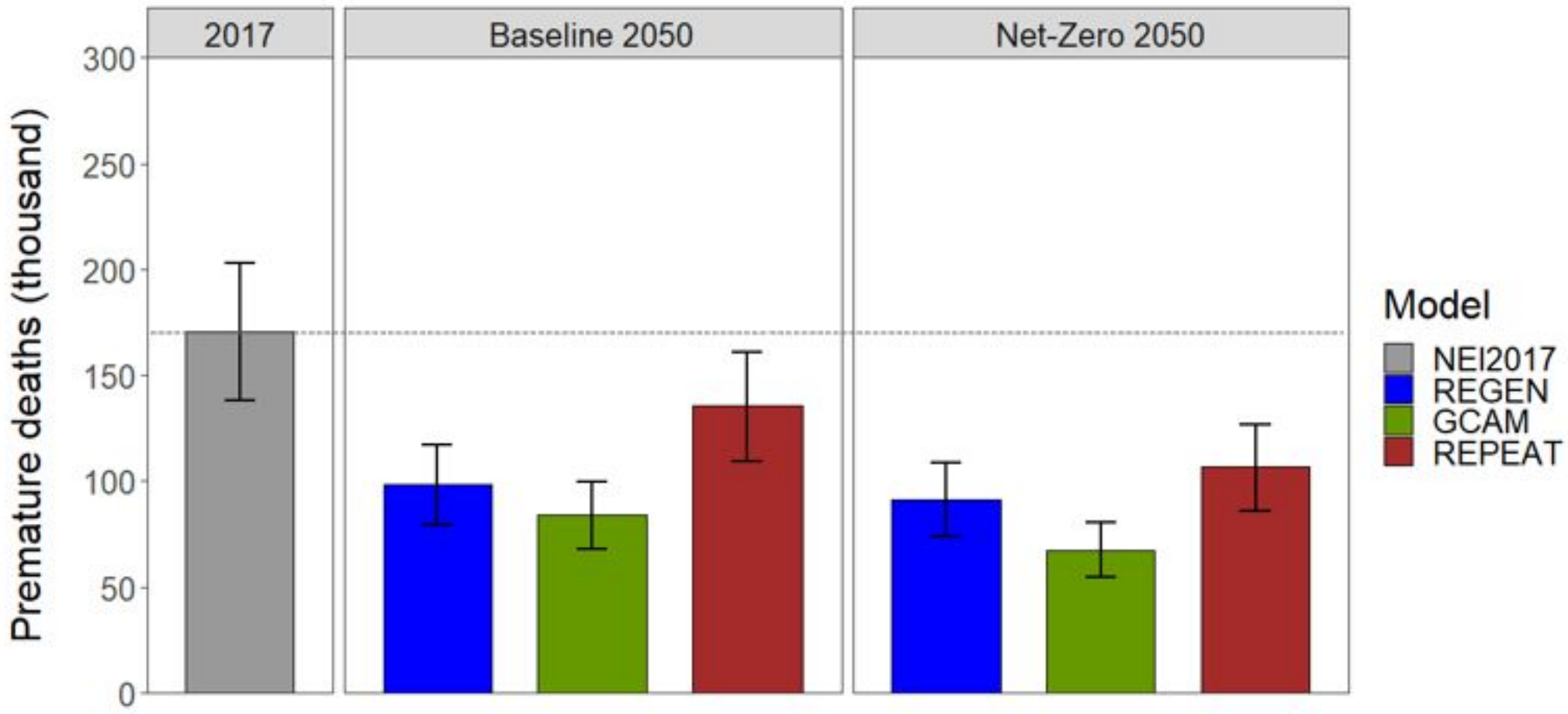

Figure S9. National health results using alternative relative risk function (RR). Bars show national $PM_{2.5}$-attributable deaths in 2017, Baseline 2050, and Net-Zero 2050 using the Global Exposure Mortality Model (GEMM) as an alternative exposure–response function. Error bars indicate 95% CI in the relative risk estimate.

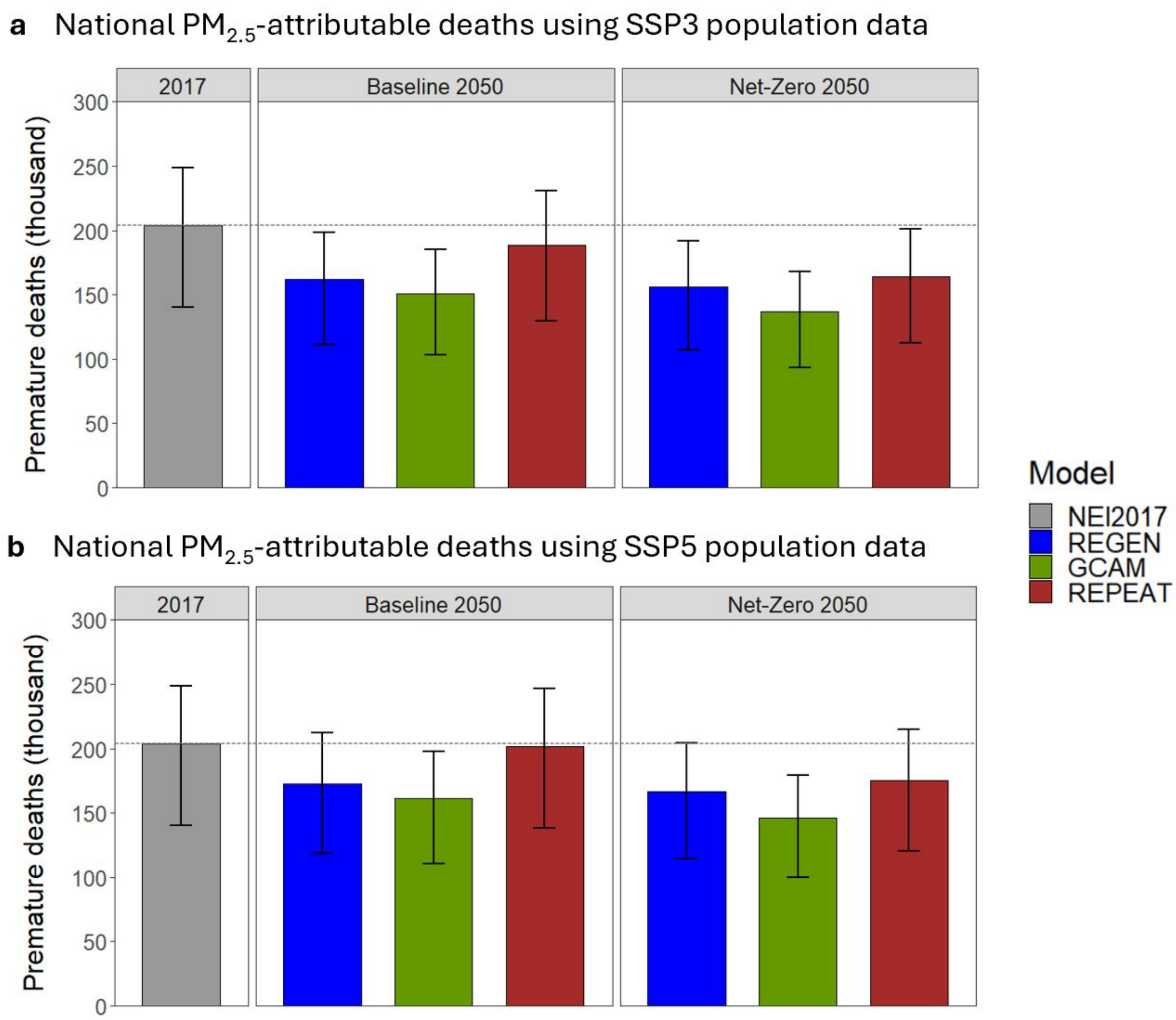

Figure S10. National health results using alternative SSP assumptions for population. Panels show national $PM_{2.5}$-attributable deaths under 2017, Baseline 2050, and Net-Zero 2050 using alternative population projections: (a) SSP3 and (b) SSP5. The main analysis uses SSP2 population assumptions. Error bars indicate 95% CI in the relative risk function. These sensitivity results evaluate how alternative demographic assumptions affect the magnitude of estimated $PM_{2.5}$-attributable mortality.

Reference

1. Luo, H. et al. Modelling the impacts of policy sequencing on energy decarbonization. Nat. Clim. Chang. **16**, 43–51 (2026).
